# Combined loss of brevican, neurocan, tenascin-C and tenascin-R leads to impaired fear retrieval due to perineuronal net loss

**DOI:** 10.1038/s41598-025-89580-2

**Published:** 2025-02-14

**Authors:** Cornelius Mueller-Buehl, Johanna Pakusch, Verian Bader, Konstanze F. Winklhofer, Melanie D. Mark, Andreas Faissner

**Affiliations:** 1 Department of Cell Morphology and Molecular Neurobiology, Faculty of Biology and Biotechnology, Ruhr University Bochum, D-44780 Bochum, Germany; 2 Behavioral Neuroscience, Faculty of Biology and Biotechnology, Ruhr-University Bochum, D- 44780 Bochum, Germany; 3 Department Molecular Cell Biology, Institute of Biochemistry and Pathobiochemistry, Ruhr University Bochum, D-44780 Bochum, Germany; 4 Cluster of Excellence RESOLV, D-44780 Bochum, Germany; 5 Department Biochemistry of Neurodegenerative Diseases, Institute of Biochemistry and Pathobiochemistry, Ruhr University Bochum, D-44780 Bochum, Germany

**Keywords:** Fear conditioning, Molecular neuroscience, Post-traumatic stress disorder

## Abstract

In conditions such as neurodegenerative diseases, posttraumatic stress disorder (PTSD), addiction and spinal cord injuries, restricted synaptic plasticity hinders the formation of new neuronal connections, preventing the compensation and treatment of adverse behaviors. Perineuronal nets (PNNs) significantly restrict synaptic plasticity by inhibiting synapse formation. The digestion of PNNs has been associated with short-term cognitive improvements and reduced long-term memory, offering potential therapeutic benefits in PTSD. This study investigates the correlation between PNNs and fear memory processes in extracellular matrix (ECM) mutant mice, particularly focusing on the amygdala-medial prefrontal cortex (mPFC) circuit, which is crucial for fear memory generation and maintenance. Fear conditioning was conducted on mice lacking four key ECM-molecules: brevican, neurocan, tenascin-C and tenascin-R (4x KO). These mice exhibited severe impairments in memory consolidation, as evident by their inability to retrieve previously learned fear memories, coupled with reduced PNN density and disturbed synaptic integrity along their PNNs. Additionally, changes in neural activity in the basolateral amygdala (BL) and reductions in VGAT^+^ synaptic puncta in the amygdala-mPFC circuit were observed. In contrast, tenascin single KOs showed intact fear behavior and memory compared to their control groups. Impaired fear memory consolidation can be advantageous in certain conditions, such as PTSD, making the 4x KO mice an intriguing model for future fear conditioning studies and highlighting brevican, neurocan, Tnc, and Tnr as compelling targets for further investigation. This study underscores the significance of ECM regulation for synaptic organization and the potential of PNN modulation as a therapeutic target for fear memory-related conditions.

**Supplementary Information:**

The online version contains supplementary material available at 10.1038/s41598-025-89580-2.

## Introduction

Psychiatric diseases impose a significant burden on society, affecting both individuals and their social environment. In 2019, approximately 970 million people worldwide were living with a mental disorder^1^. Since the physiological alterations in these neurological diseases are difficult to detect, and in many cases unknown, proper treatment is problematic. Most research in this regard concentrates on neural networks and/or neurotransmission^2–4^. While some treatments provide symptomatic relief, no cure or clear physiological biomarkers for early detection have been identified^5^.

In the last decade, the involvement of the ECM in neuropsychiatric and neurodegenerative research has increased^6,7^. Alterations in a specialized ECM structure called the perineuronal net (PNN) have been found in various neuropsychiatric diseases such as schizophrenia, bipolar disorder, addiction, and posttraumatic stress disorder (PTSD)^8–12^. Notably, manipulating these PNNs led to significant cognitive improvements under pathological conditions^13^. In the future, alterations of PNNs may serve as predictors for the onset of these psychiatric disorders, and manipulation of the ECM may be utilized as a therapy to enhance cognitive abilities.

PNNs envelop a subset of neurons, mainly parvalbumin-positive interneurons, in the central nervous system in a netlike manner around the soma and proximal dendrites^14,15^. In general, PNNs consist of a hyaluronan backbone and link-proteins of the HAPLN family, lecticans, and tenascin-R^16–20^. There is also a common basic framework of PNNs that may contain a heterogeneous composition depending on the brain area and the type of neuron they envelop^21–23^. Therefore, the neural role of individual PNN components and compositions is essential to analyze in the context of neuropsychiatric diseases. A well-established method to study anxiety and fear is classical Pavlovian fear conditioning (FC)^24^. Fear conditioning can be used as a model for PTSD and, in general, for fear memory and learning processes^25^. In FC a conditioned and an unconditioned stimulus are coupled to elicit a conditioned response^26–28^ that relies on a coordinated network of specific brain regions across fear learning stages. The amygdala and its subnuclei play a crucial role in the initial phase of acquisition. The lateral amygdala (LA) acts as a hub for the CS and US stimuli^29^ and conveys input information via projection neurons to the basolateral amygdala (BL) and the central nucleus of the amygdala (CeA). Neurons located in the LA and their projections to other brain regions are critical for the acquisition of fear^30^ as well as for the retrieval of previously learned fear memories^31–33^. In contrast to the LA, the medial prefrontal cortex (mPFC) is considered a memory engram storage and encoding unit during fear extinction learning^34^. However, during fear retrieval, the number of active neurons in the prelimbic cortex (PrL), a subregion of the mPFC, increases^35,36^. The PrL region is an important structure within the mPFC for learned fears, as it integrates information from auditory and contextual inputs, and regulates the expression of fear memories through its projections to the amygdala nuclei^37^. In 2009, Gogolla et al. demonstrated that PNN degradation by chondroitinase ABC (ChABC) in the BL can reduce fear responses following extinction training^38^. However, only about 2% of chondroitin sulfate proteoglycans (CSPGs) are bound to PNNs, with the majority unbound in the extracellular space^39^. Consequently, digestion by ChABC is not PNN-specific, and assessing the role of individual PNN components during fear memory is challenging. Studies in global KO models of core CSPGs or PNN-associated and regulating components, such as tenascin-C and tenascin-R, regarding associative fear learning are relatively sparse. Mice lacking the core CSPG aggrecan were able to relearn after undergoing a synapse withdrawal learning paradigm^40^, whereas the loss of brevican resulted in a slight decrease in working memory without changes in anxiety^41^. Global KO of tenascin-R provided inconclusive results, with increased anxiety in the open field and elevated plus maze tests^42^, and decreased anxiety in the open field^43^. Tenascin-C KO animals displayed normal contextual fear acquisition and retrieval, whereas extinction learning, and recall were impaired^44^.

To date, few studies have been conducted using ECM KO models, specifically examining possible synergistic effects when targeting multiple PNN components. In this study, we used a 4x KO model to investigate the possible effects of PNNs on fear learning and memory in a Pavlovian fear conditioning paradigm. In addition, we selectively examined the effects of Tnr and Tnc separately to gain further insight into these PNN-regulating components. To gain insights into the neural circuitry driving PNN fear learning and memory, we examined changes in neuronal activity in the amygdala and medial prefrontal cortex, as well as the PNN structure and distribution of excitatory and inhibitory presynapses along these PNNs in brain areas. Given that the ECM plays a crucial role in fear memory formation, it is important to understand the specific roles of individual PNN components such as Tnr and PNN-associated molecules like Tnc in the context of fear memory. In the future, manipulations targeting specific components could potentially alleviate everyday difficulties for individuals with PTSD.

## Materials and methods

### Animals

Behavioral testing was conducted in 12–14-week-old male mice. Animals were kept in groups of two to three per cage with unlimited access to food and water. Mice were isolated from the central mouse colony in a temperature-controlled room with a 12-h light-dark cycle. Cage changes and handling of mice before behavioral testing were performed by the responsible scientist. The last cage change was at least three days prior to the start of the fear conditioning paradigm to reduce additional stress before behavioral testing. The experiments were conducted during the light phase in five separate fear conditioning sessions. Testing was conducted in 4x KO mice, created by Rauch et al. through crossbreeding single KO mouse lines, which incorporate the transgenic KO constructs for brevican, neurocan, Tenascin-C and Tenascin-R^45–49^. A precise description of the genetic background and knockout construct was recently published^50^. 129S2/SvPasCrl mice (Charles River Laboratories strain code 287, further referred to as 4x WT) served as a control. Single tenascin-C (Tnc) KO and tenascin-R (Tnr) KO mice, carrying only the Tnc respectively the Tnr construct, and their corresponding background strains (Tnc WT and Tnr WT) as controls were also examined. Mouse colonies were housed at the animal facility of the Faculty of Biology and Biotechnology, Ruhr University Bochum (Bochum, Germany). Experiments were authorized by the local ethics committee (Bezirksamt Arnsberg) and the animal care committee of Nordrhein-Westfalen (LANUV; Landesamt für Umweltschutz, Naturschutz und Verbraucherschutz Nordrhein-Westfalen, Germany). The studies were conducted following the European Communities Council Directive of 2010 (2010/63/ EU) for the care of laboratory animals and supervised by the Animal Welfare Committee of Ruhr-University Bochum and conducted in compliance with the ARRIVE guidelines.

### Fear conditioning paradigm

The mice were handled daily prior to the behavioral tests to minimize stress responses. Specifically, each mouse was individually handled until it no longer exhibited a fleeing reaction when the home cage was opened or when a hand was placed inside. The duration of handling was adjusted based on each mouse’s behavior, but as a general guideline, mice were handled for approximately 5 min per day over 5 consecutive days. During this time, efforts were focused on habituating the mice to remain calm during removal from their home cage using a paper roll.

Acquisition and extinction training were conducted in two contexts (A and B), which were achieved by changing the light, texture, odor and visual surroundings to achieve fear conditioning in response to an auditory cue and not the context (*n* = 5–6/group). A fear conditioning chamber with black and white striped walls and a darkened experimental room were contained in context A. A footshock grid was inserted at the base of the chamber. The chamber was scented with 0.05% Helipur^®^ (B. Braun SE, Melsungen, Germany) and illuminated with infrared and green light (65 lx). Context B incorporated a brightly lit experimental room, whereas the fear conditioning chamber was equipped with infrared lighting (outside the perceivable vision spectrum of mice) to enable video recording, gray walls, texturized flooring and 70% EtOH to ensure proper context change. The fear conditioning chamber was built in-house and consisted of an acrylic glass chamber (23 × 25 × 24 cm). The chamber was placed inside a noise-reducing cabinet that reduced the background noise inside the testing chamber to 35 ± 5 dB. During behavioral testing, mice were recorded using a video camera (Mako U-130B Allied Vision Technologies GmbH, Stradtroda, Germany) for post-hoc analysis. For sound administration a speaker (FR 58; VISATON GmbH & Co. KG Haan, Germany) was mounted centrally above the chamber. Sound and shock deliveries were controlled using a custom-written MATLAB script (The MathWorks Inc., Natick, MA). On day one, mice were placed into the conditioning chamber in context A and underwent fear acquisition, which consisted of a 2 min baseline period, followed by 10 tones (CS 30 s, 7.5 kHz, 60 dB) which were co-terminated with an electric footshock (US 2 s, 0.45 mA). The inter-trial interval (ITI) varied between 20 and 180 s. After each animal, the chamber was carefully cleaned using soap and water. The mice were brought to context B for fear retrieval after a 24-h consolidation period. One tone (CS 60 s, 7.5 kHz, 60 dB) was applied after a baseline period of 2 min.

An extended fear paradigm was also utilized in which mice were subjected to an additional extinction learning period. The protocol included five subsequent days of extinction after completing the previously described protocol. Each extinction session, conducted in context B, consisted of 15, 30 s long tones (7.5 kHz, 60 dB), with an inter-trial interval ranging from 20 to 120 s.

### Behavior evaluation

EthoVision XT 11.5 (Noldus Information Technology BV, Wageningen, Netherlands) was used to investigate the freezing behavior. Freezing was defined as the absence of movement except for respiratory movement. For fear acquisition, freezing was analyzed during the 30 s time interval of CS presentation. The analysis of the retrieval session contained the 120 s baseline period and the 60 s CS presentation. The analysis settings were chosen to reflect freezing following the guidelines described above. Freezing was determined by a less than 0.02% change in pixels from one frame to the next for a minimum of 2 s. The videos were recorded at a rate of 49.76 frames/s, and a threshold of 7 was set for activity, along with a noise filter of 1 and a filter to remove compression artifacts. The occurrence of freezing behavior was manually confirmed. Velocity analysis was also performed with EthoVision XT 11.5 for baseline and cue retrieval by automatic video tracking of the animal in the arena. Proper tracking of the animals was manually verified and corrected. Velocity was calculated by dividing the distance moved during the 120 s baseline period or during the 60 s cue retrieval period by the corresponding time period.

### Immunohistochemistry

Tissue samples were collected 90 min following the behavioral studies (*n* = 5–6/group). Animals were euthanized by cervical dislocation. The brains of 4x WT, 4x KO, Tnc WT, Tnc KO, Tnr WT and Tnr KO mice were initially fixed in 4% paraformaldehyde (PFA), cryoprotected, and finally embedded in Tissue-Tek freezing medium (Thermo Fisher Scientific, Cheshire, United Kingdom). Subsequently, for cell counting, basic quantification purposes, assessment of PNN structure and to detect synaptic proteins, the brain tissue was sectioned sagittally (40 μm thick) using a cryostat (CM3050 S, Leica) set at coordinates for LA (lateral: 3.44 mm), for BL (lateral: 2.40 mm) and PrL (lateral: 0.12 mm). Ex vivo slices, free-floating sections were prepared. For the free-floating staining process, tissue sections were first incubated in 1× PBS for 20 min and then blocked using a solution containing 10% (v/v) normal goat serum (Dianova, Hamburg, Germany), 1% w/v BSA, and 0.1% (v/v) Triton-X-100 in 1× PBS for 1 h at room temperature. Primary antibodies (Table S1) were diluted in blocking solution and incubated at 4 °C for 3 days. Tissue sections were then washed three times with 1× PBS for 30 min each and incubated with the appropriate secondary antibodies for 2 h.

### Fluorescence microscopy

For counting cFOS ,*Wisteria floribunda* agglutinin (WFA) and parvalbumin positive cells, sagittal brain sections (*n* = 5–6/group) were recorded with a fluorescence stereo microscope (Axio Zoom.V16, Zeiss, Göttingen, Germany). 1.12 mm x 895.11 μm large areas of the PrL, BL and LA were selected. The number of positive cells was counted using the cell counter tool of the ImageJ software (ImageJ 1.51w, National Institutes of Health; Bethesda, MD, USA). To evaluate the mean intensity of cFOS^+^ cells, the CellProfiler 4.2.1 software (Broad Institute, Cambridge, MA, USA) was used. Images were first converted from color to grayscale. cFOS^+^ cells were identified with the “IdentifyPrimaryObjects” module, specifying a “typical diameter of object in pixel units” ranging from 15 to 40 pixels. An “adaptive threshold strategy” was applied using the “otsu thresholding method,” along with “three-class thresholding”. The threshold bounds were set to 0.2 and 1.0, respectively. Finally, the “measure object intensity” module was used to calculate the mean intensity of the identified cells.

### Visualization of PNN structure

Immunohistochemical staining with WFA (PNN marker) was performed using fluorescence super-resolution structured illumination microscopy on a Zeiss Elyra PS.1 plus LSM880 microscope. Z-stack imaging was used to acquire a stack of 30–60 sections with an interval of 0.3 μm, depending on the genotype and brain area. Laser power and gain were kept constant during all measurements.

To analyze the PNNs, SIM images were imported into IMARIS 9.3.1, a software for 3D surface rendering and quantitative analysis. The “create surface” tool was used to manually draw a surface containing the WFA-positive PNN for each optical section. A threshold was chosen to exclude background signal. A 3D surface containing a single PNN-enwrapped neuron was generated and defined as a region of interest (ROI). WFA-positive signal outside the ROI was suppressed. The volume of the generated ROI reflects the volume of the PNN. To determine the density of the PNN, a 3D surface of the WFA-positive signal inside the ROI was generated and its volume was automatically measured. By dividing the isolated WFA positive volume by the overall PNN volume, the amount of WFA-positive signal around the neuron was identified and represented as a percentage. The WFA total fluorescence intensity of every PNN was also determined via IMARIS, and the intensity values were normalized to the mean intensity value of the wild type. For each group, 15 PNN-enwrapped cells were analyzed (Figure S2).

### Visualization of PNN-associated excitatory and inhibitory presynapses

The distribution of excitatory and inhibitory presynapses along PNNs in the PrL, BL, and LA of both WT and ECM-KO mice was examined by immunohistochemical staining using WFA and specific antibodies against VGAT or VGLUT1. Three-dimensional (3D) surfaces representing WFA-positive PNNs were generated. The “spots” analysis tool from the IMARIS software was used to analyze the presynaptic puncta distribution at the PNN, with a minimum synaptic puncta diameter of 0.2 μm. Background noise was removed using the “background subtraction” tool from the IMARIS software with the same settings in all examined PNNs. The synaptic distribution on a certain number of PNN-enwrapped cells was compared between the WT and ECM-KO groups.

### Data visualization and statistical analysis

Graphs were created with GraphPad Prism (GraphPad Software, San Diego, California, USA, www.graphpad.com) and post-processed with CorelDraw^®^ Graphics Suite (Corel Corporation Ottawa, Canada). Data are presented as tukey boxplots with the exception of fear acquisition behavior, which is plotted as the mean, with the ± SEM as the shaded area. Fear behavior was analyzed by two-way repeated-measures Analysis of Variance (two-way RM ANOVA), as implemented in GraphPad, to identify significant changes in freezing behavior over the course of the acquisition trials and between genotypes, as well as the interaction between genotypes and trials. Geisser-Greenhouse correction (GGC) was applied. Post-hoc Sidak-corrected analysis was performed if the interaction effect was *p* < 0.05. Differences in freezing and velocity between genotypes during baseline and retrieval were also examined using two-way RM ANOVA with GGC, followed by post-hoc Tukey´s multiple comparison test. For histological analysis of the number of cFOS and WFA positive cells, the cell counts, intensity measurements, PNN structure and synaptic distribution were tested with the Kolmogorov-Smirnov test on normality distribution. Data that passed the normality test, was then tested with One-way ANOVA (One-way analysis of variance) and by post-hoc Tukey´s multiple comparison test. Data that did not pass normality tests, were analyzed via Mann-Whitney test. Statistical significance was set at *p* < 0.05.

## Results

### Loss of tenascin R, tenascin C, brevican and neurocan leads to fear memory deficits

**Fig. 1:**
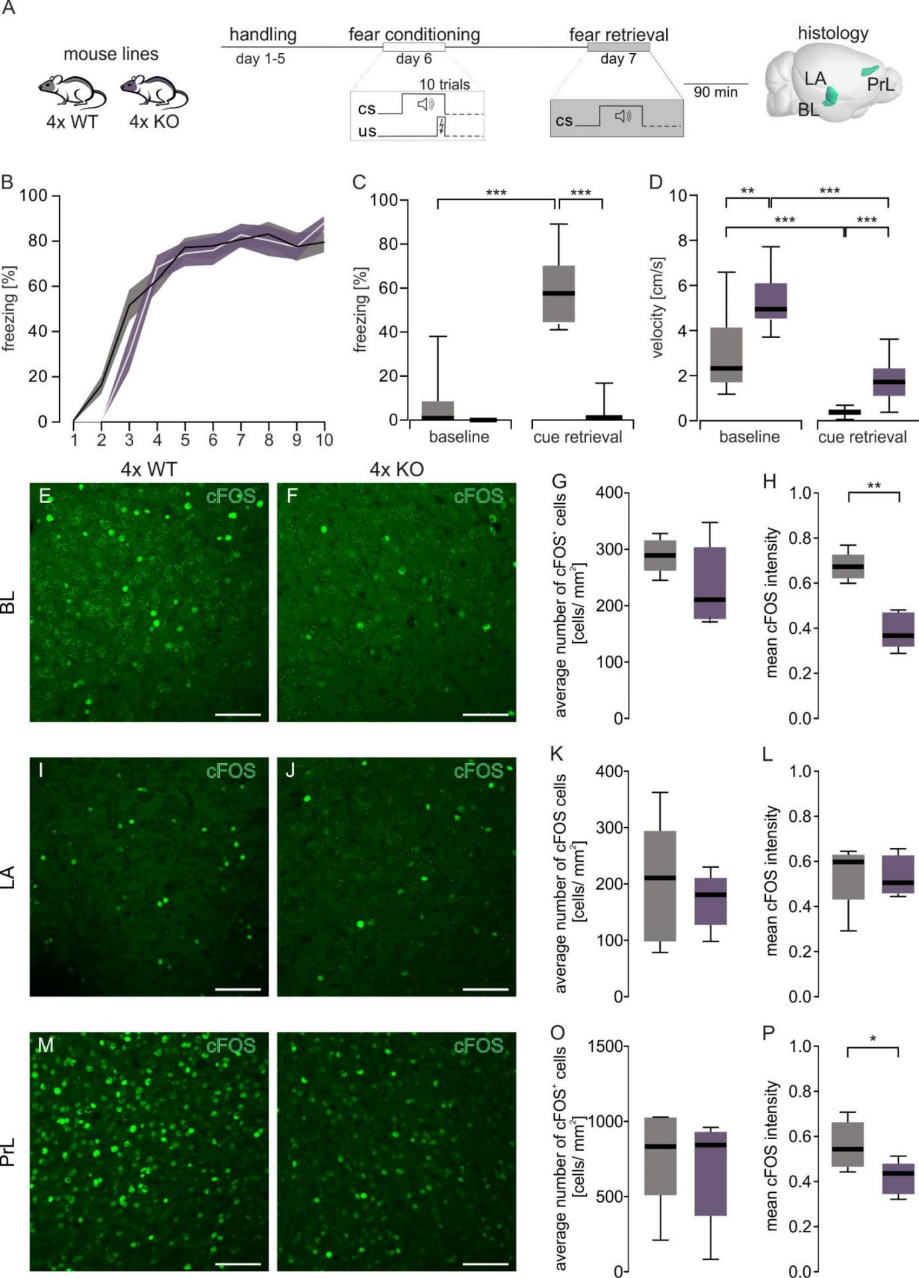
Fear retrieval deficits in mice deficient in tenascin C, tenascin R, brevican and neurocan. (**A**) Schematic representation of the auditory fear conditioning protocol consisting of a fear acquisition phase where ten tone and footshock pairings were applied, followed by a retrieval session consisting of a baseline period of 120 s and a cue retrieval phase of 60 s the next day in context B. (**B**) Fear acquisition of 4x KO and control 4x WT mice displayed as percentage of the time spent freezing during the individual tones. Data are shown as mean (line) ± SEM (shaded area). The number of animals tested is shown in parentheses behind the genotype description. (**C**) Comparisons of the time spent freezing during the 120 s baseline and 60 s cue retrieval for 4x KO mice and their corresponding control show significant differences between baseline and retrieval freezing for 4x WT but not for 4x KO mice with significant differences between genotypes (detailed statistics in Table S3). (**D**) Further analysis of the behavior during baseline and cue retrieval revealed significant decreases in velocity between baseline and cue retrieval for both genotypes with additional differences between genotypes during baseline and retrieval. (**E & F**, ** I & J**, **M & N**) Representative images of cFOS^+^ cells (green) in BL, LA and PrL of 4x WT and 4x KO mice during cue retrieval. **(G**,** K**,** O)** The number of cFOS^+^ cells is comparable between 4x WT and 4x KO in all analyzed regions. (**H**,** L**,** P**) The mean intensity is significantly reduced in the BL and PrL of 4x KO mice in comparison to 4x WT mice, but comparable in LA between both genotypes. BL, basolateral amygdala; cFOS, cellular FBJ murine osteosarcoma viral oncogene homolog; LA, lateral amygdala; PrL, prelimbic cortex; **p < 0.01; *p < 0.05; *N* = 5. scale bar = 200 μm.

In the current study we investigated the role of the PNN in fear learning and memory by utilizing a 4x KO mouse model with a selective KO of four PNN components, brevican, neurocan, tenascin-C, and tenascin-R. 4x KO and 4x WT littermates were subjected to paired tone and shock presentations in a classical fear conditioning paradigm and subsequently immunohistochemically examined in brain structures relevant for fear memory (Fig. 1A). Throughout the trials (Fig. 1B) both genotypes showed increasing fear responses (*F*_(4.378, 113.8)_ = 93.60. *p* ≤ 0.001, two-way RM ANOVA GGC). More specifically, an interaction effect between genotype and trial was observed (*F*_(9, 234)_ = 1.936, *p =* 0.048, two-way RM ANOVA GGC). At the beginning of the fear acquisition session, 4x KO mice displayed a delayed increase (trial two post-hoc Sidak-corrected t_(13)_ = 4.188 *p* = 0.011) in fear responses that subsided at the end compared to the 4x WT group.

To test whether the mice displayed conditioned fear behavior 24 h after fear acquisition, we examined cue retrieval in a novel context. There was an interaction effect between genotype (4x KO vs. 4x WT) and trial (baseline vs. retrieval), indicating that the loss of PNN components plays a role in fear retrieval (Fig. 1C *F*_(1, 13)_ = 644.3, *p* ≤ 0.001, two-way RM ANOVA GGC). To examine this interaction effect in more detail, post hoc analysis was performed and revealed no significant difference in baseline freezing between the two groups. While 4x KO mice showed no significant increase (mean freezing 2.21%, post-hoc Tukey test *p* = 0.343), 4x WT mice displayed higher (mean freezing 58.45%, post-hoc Tukey test *p* ≤ 0.001) fear behavior during cue retrieval compared to baseline. As the interaction effect implies, 4x KO mice exhibited reduced freezing compared to 4x WT mice, indicating that the loss of PNN components diminished the conditioned fear response during fear retrieval (post-hoc Tukey test *p* ≤ 0.001).

Although 4x KO mice did not show significant fear learning during retrieval, they showed an overall decrease in movement (Fig. 1B). To reflect this, the velocities of both groups were analyzed during retrieval and at baseline (Fig. 1D). Similar to the freezing behavior, there was a significant interaction effect (*F*_(1, 13)_ = 5.414, *p =* 0.037, two-way RM ANOVA GGC, Table S4). Contrary to the fear behavior, the velocity of 4x KO mice during retrieval was significantly decreased compared to baseline (post-hoc Tukey test *p* ≤ 0.001). This was apparent not only for the 4x KO group but also consistent with findings in freezing responses in the 4x WT group (post-hoc Tukey test *p* ≤ 0.001). Although there was a reduction in movement in the 4x KO mice suggesting the ability to recognize the CS during retrieval, when compared to the movement of the 4x WT clearly shows a deficit in fear retrieval responses (post-hoc Tukey test *p* ≤ 0.001).

To further investigate the retrieval effect, an extended fear conditioning paradigm that included extinction training was used to examine whether differences in retrieval persisted during extinction training. Although fear responses were elevated during acquisition (Figure S8; F_(3.751, 37.51)_ = 35.99, *p* ≤ 0.001, two-way RM ANOVA GGC) in the 4x KO mice, the previously observed effect during retrieval persisted throughout extinction. There was a significant difference in freezing levels between the two genotypes during this phase, with the 4x KO mice exhibiting lower freezing levels compared to the 4x WT mice (F_(1, 10)_ = 22.38, *p* ≤ 0.001, two-way RM ANOVA GGC) during extinction. However, there was no detectable change of freezing responses during extinction within the genotypes. (Trial F_(4.574, 45.74)_ = 1.296, *p* ≤ 0.284, two-way RM ANOVA GGC). During subsequent extinction sessions, fear extinction training resulted in augmented freezing responses within sessions (extinction sessions II, III, and V; all p-values below 0.011; two-way RM ANOVA GGC), while sustained genotype differences were observed (Figure S8, Table S5 all p-values below 0.008; two-way RM ANOVA GGC).

Previous studies have shown that PNNs regulate neuronal activity and may affect the downstream behavior of an organism^40,51^. To determine whether there is a correlation between cue retrieval response observed in the 4x KO mice and changes in neural activity within fear consolidation-associated brain regions, cFOS expression levels in the BL, LA, and PrL were investigated. Tissue samples from both 4x KO and 4x WT mice were collected 90 min after the cue retrieval and stained with an antibody against cFOS (Fig. 1E&F, I&J, M&N). To precisely locate the brain areas to be examined, overview images were initially taken for orientation in the sagittal brain sections (Figure S1).

Notably, no differences in cFOS^+^ cells between the two mouse groups were detected in the fear associated brain areas investigated (BL, LA, and PrL) (Fig. 1G, K, O and Table S6).

Since the number of cFOS^+^ cells does not describe the strength of their activity, cFOS intensities were also measured to investigate the possible influence of 4x KO on neuronal networks. No differences in the cFOS intensity were detected in LA between 4x WT and 4x KO (Fig. 1L, Table S6). In contrast, in the BL (4x WT 0.67 ± 0.02 mean intensity vs. 4x KO 0.39 ± 0.04 mean intensity; *p* ≤ 0.01; Fig. 1H), as well as in the PrL (4x WT 0.56 ± 0.05 mean intensity vs. 4x KO 0.42 ± 0.07 mean intensity; *p* ≤ 0.05; Fig. 1P) the average intensity of cFOS expression was reduced in the 4x KO during cue retrieval. The number of cFOS and WFA double positive cells was also significantly reduced in the BL of 4x KO mice, indicating a link between the KO of PNN components and alterations in neuronal activity during cue retrieval (4x WT 27.4 ± 4.37 cFOS^+^ cells/mm^2^ vs. 4x KO 16.7 ± 1.20 cFOS^+^ cells/mm^2^; *p* ≤ 0.05; Figure S3C), where the number of WFA and cFOS double positive cells in the LA and PrL remained comparable. As a control for the c-FOS^+^ cells, immunohistochemical staining was preliminarily performed on brain slices of wild-type mice that had not been subjected to a fear conditioning test (data not shown). In these animals, c-FOS expression was examined in the three regions of interest, revealing almost no positive cells in Bl and La. However, c-FOS^+^ signals were observed in the mPFC. The signal did not exhibit the same intensity as c-FOS-positive cells following FC. Consequently, it cannot be conclusively stated that all neuronally active cells in this region were activated by FC. Nevertheless, the experiment provides a solid overview of the overall neuronal activity in these areas after FC. Since the 4x KO mice exhibited a successful fear response during fear acquisition but did not show a freezing response to the conditioned tone on the subsequent retrieval day, this indicates a deficit in their consolidation of fear memories. However, they did display reduced velocity when subjected to a conditioning stimulus. Additionally, the KO of ECM molecules influenced neuronal activity in brain regions, BL and PrL, which are associated with fear consolidation. Our results show a reduction in the number of PNN-encased active neurons, consequently, a reduction in the overall activity of the neuronal network in the BL which underscores the role of the PNN in fear memory processing.

### Normal fear behavior in tenascin-C and tenascin-R single KO mice

**Fig. 2:**
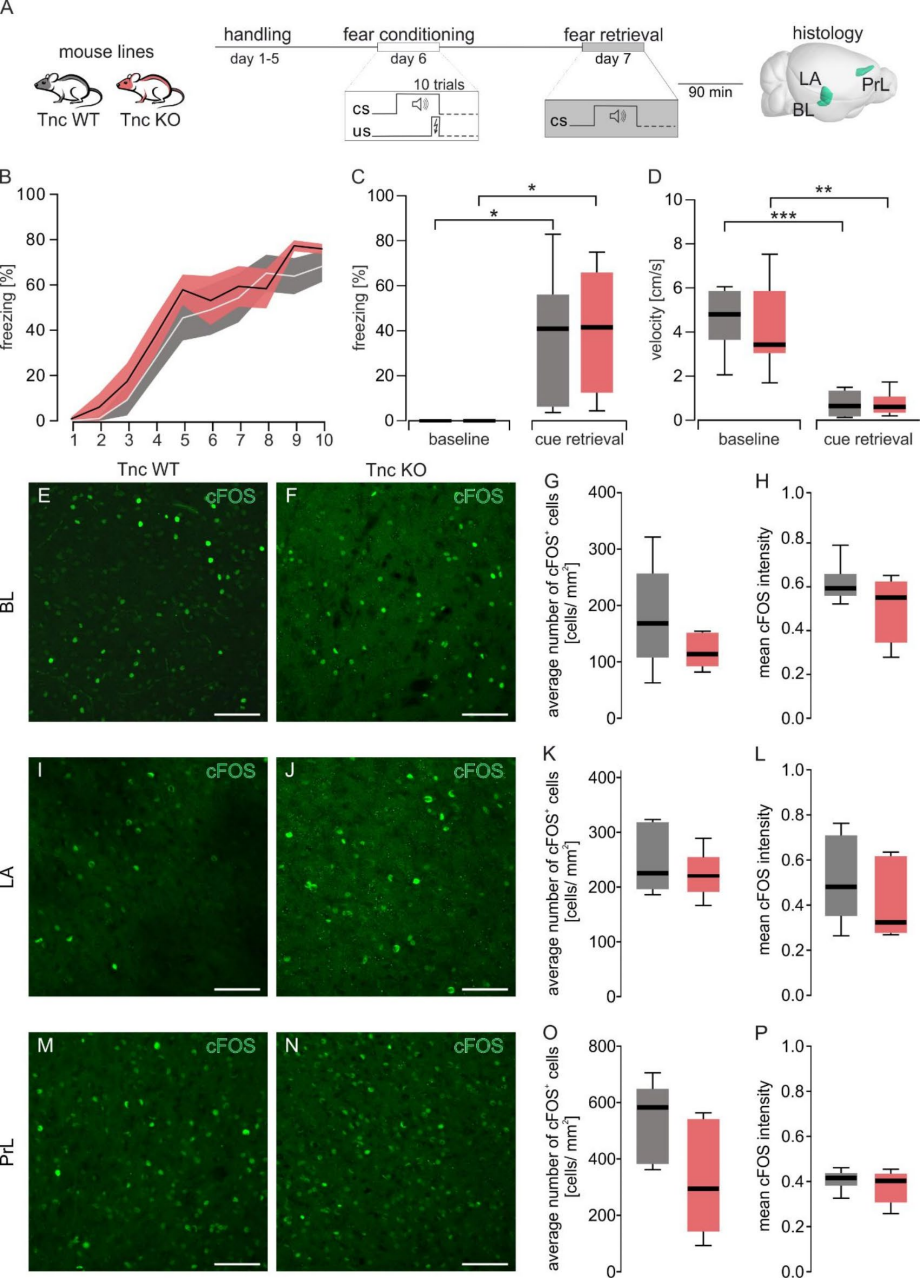
Tenascin C deficient mice exhibited no fear retrieval deficits. (**A**) Schematic representation of the auditory fear conditioning protocol consisting of a fear acquisition phase where ten tone and footshock pairings were given, followed by a retrieval session consisting of a baseline period of 120 s and a cue retrieval phase of 60 s on the next day in context B (**B**) Fear acquisition of Tnc KO and control mice where the tone was coupled to a footshock. Percentage of time spent freezing during the tone. Data are shown as mean (line) ± SEM (shaded area). The number of animals tested is shown in parentheses behind the genotype description. (**C**) Comparison of the time spent freezing during the 120 s baseline and 60 s cue retrieval for tenascin-C and their corresponding controls showed significant differences between baseline and retrieval freezing (detailed statistics in Table S4). **(D)** Further analysis of the behavior during baseline and cue retrieval revealed significant decreases in velocity between the baseline and cue retrieval (detailed statistics in Table S5). (**E & F**, ** I & J**, **M & N**) Representative images of cFOS^+^ cells (green) in BL, LA and PrL of Tnc WT and Tnc KO mice during cue retrieval. (**G**,** K**, **O**) The number of cFOS^+^ cells was comparable in Bl, LA and PrL of Tnc WT and Tnc KO animals. (**H**,** L**,** P**) No significant differences in mean intensity of cFOS signal between Tnc WT and Tnc KO mice. Tnc WT, tenascin-C wild-type; Tnc KO, tenascin-C KO; *N* = 5–6. scale bar = 200 μm.

**Fig. 3:**
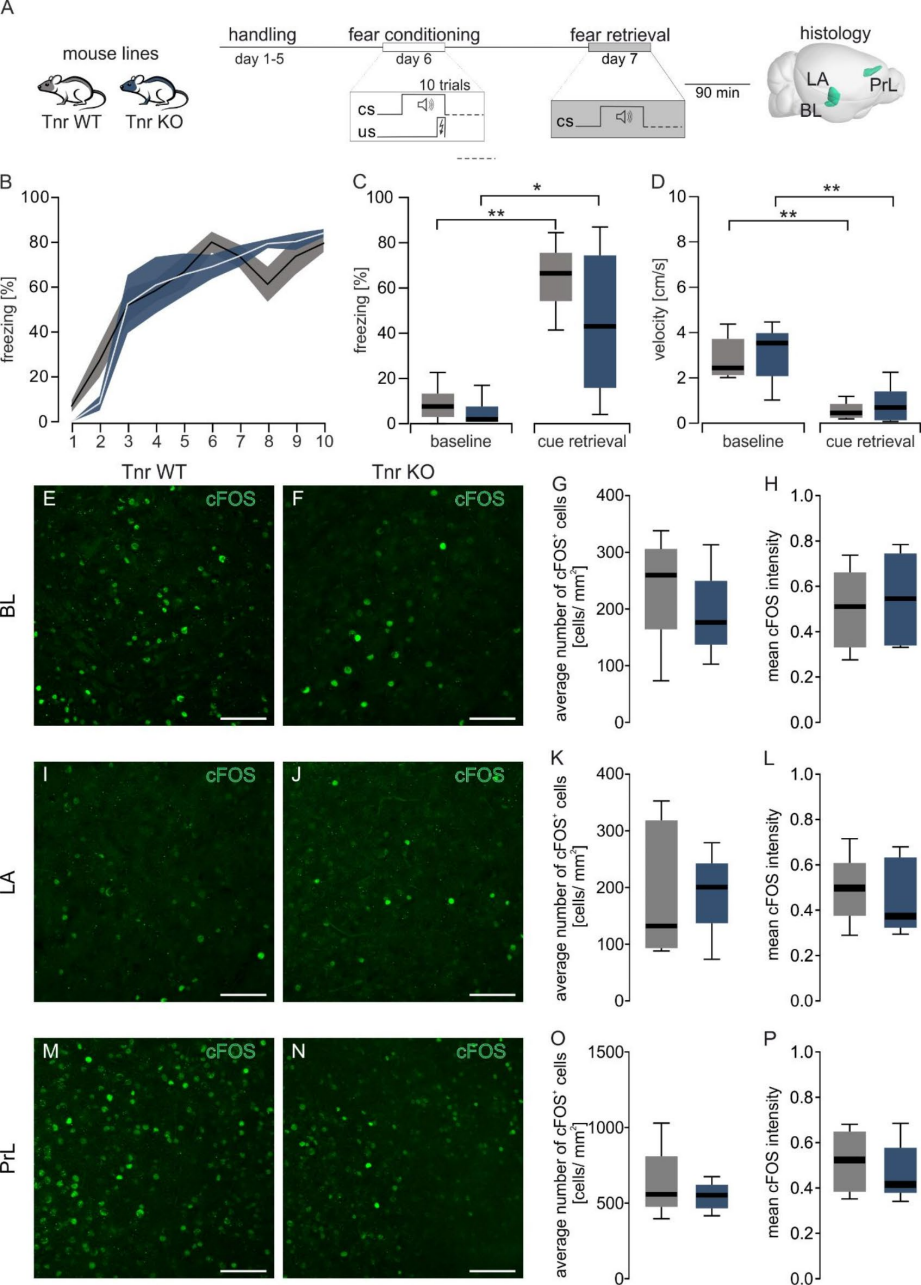
Tenascin R deficient mice demonstrated no fear retrieval deficits. (**A**) Schematic representation of the auditory fear conditioning protocol consisting of a fear acquisition phase where ten tone and footshock pairings were given, followed by a retrieval session consisting of a baseline period of 120 s and a cue retrieval phase of 60 s on the next day in context B. (**B**) Fear acquisition of Tnr KO and control mice where the tone was coupled to a footshock. Percentage of time spent freezing during the tone. Data are shown as mean (line) ± SEM (shaded area). The number of animals tested is shown in parentheses behind the genotype description. (**C**) Comparison of the time spent freezing during the 120 s baseline and 60 s cue retrieval for tenascin-R and their corresponding controls showed significant differences between baseline and retrieval freezing (detailed statistics in Table S3). **(D)** Further analysis of the behavior during baseline and cue retrieval revealed significant decreases in velocity between baseline and cue retrieval. (**E & F**,** I & J**,** M & N**) Representative images of cFOS^+^ cells (green) in BL, LA and PrL of Tnr WT and Tnr KO mice during cue retrieval. (**G**,** K**,** O**) The number of cFOS^+^ cells was comparable in BL, LA and PrL of Tnr WT and Tnr KO animals. (**H**,** L**,** P**) No significant differences in mean intensity of cFOS signal between the Tnr WT and Tnr KO mice. Tnr WT, tenascin-R wild-type; Tnr KO, tenascin-R KO; *N* = 5–6. scale bar = 200 μm.

Previous studies partially attributed behavioral abnormalities of the 4x KO mice to the single tenascin KOs. Since there were significant changes between the 4x KO and 4x WT mice, we further determined if the deletion of a single tenascin could be responsible for the disruption of the fear memory retrieval. To achieve this, two single KO models were tested, each targeting one of the ECM molecules known to alter fear behavior (tenascin-C) and fear-related learning (tenascin-R), using the same experimental setup followed by immunohistochemical analyses in brain structures relevant for fear memory (Figs. 2A and 3A). During associative fear learning in the acquisition phase, both tenascin-KO mouse lines showed no differences in learning compared with their respective controls. Tnc KO (Fig. 2B, F_(3.695, 51.73)_ = 32.13, *p* ≤ 0.001, two-way RM ANOVA GGC) and Tnr KO (Fig. 3B, F_(2.64, 26.43)_ = 40.45, *p* ≤ 0.001, two-way RM ANOVA GGC) mice showed increased freezing responses during the acquisition phase. Associative fear memory was tested 24 h later in context B during cue-dependent fear retrieval and compared to baseline freezing. Both single tenascin KO lines and control mice froze more during retrieval than baseline (Tnc Fig. 2C *F*_(1, 5)_ = 55.46, *p* ≤ 0.001, Tnr Fig. 3C *F*_(1, 7)_ = 29.93, *p* ≤ 0.001, two-way RM ANOVA GGC for details see Table S3). Although all groups independently showed fear learning, there was no difference between the single tenascin KO and their respective controls in their fear responses within fear retrieval or baseline (both *p* > 0.180 two-way RM ANOVA GGC for details see Table S3). The rise in freezing response and reduction in movement as reflected in the mean velocity was decreased during retrieval in comparison to the baseline (Tnc *F*_(1, 7)_ = 120.4, *p* ≤ 0.001, Tnr *F*_(1, 5)_ = 97.67, *p* ≤ 0.001, two-way RM ANOVA GGC see Figs. 2D and 3D and Table S4).

In agreement with the fear learning behavior, cFOS^+^ cells and mean intensities from fear associative brain regions in the tenascin single KOs showed no differences compared to their WT controls. Both Tnc (Fig. 2E-P) and Tnr (Fig. 3E-P) single KOs exhibited comparable number and intensity of cFOS^+^ cells in the BL, LA and PrL during cue retrieval (Table S6). This unchanged neuronal activity in the brain structures associated with fear consolidation in tenascin single KO mice was consistent with normal fear behavior. Furthermore, it suggests that the KO of tenascin-C alone does not have significant effects on fear behavior. Based on these results, the single KO of tenascins alone appear not to be sufficient to alter the fear behavior in mice. The observed changes in the 4x KO animals may originate in the lectican KO or from the lack of interactions between the four ECM molecules.

### Impairments in PNN number and structure of 4x KO and Tnr KO mice

**Fig. 4:**
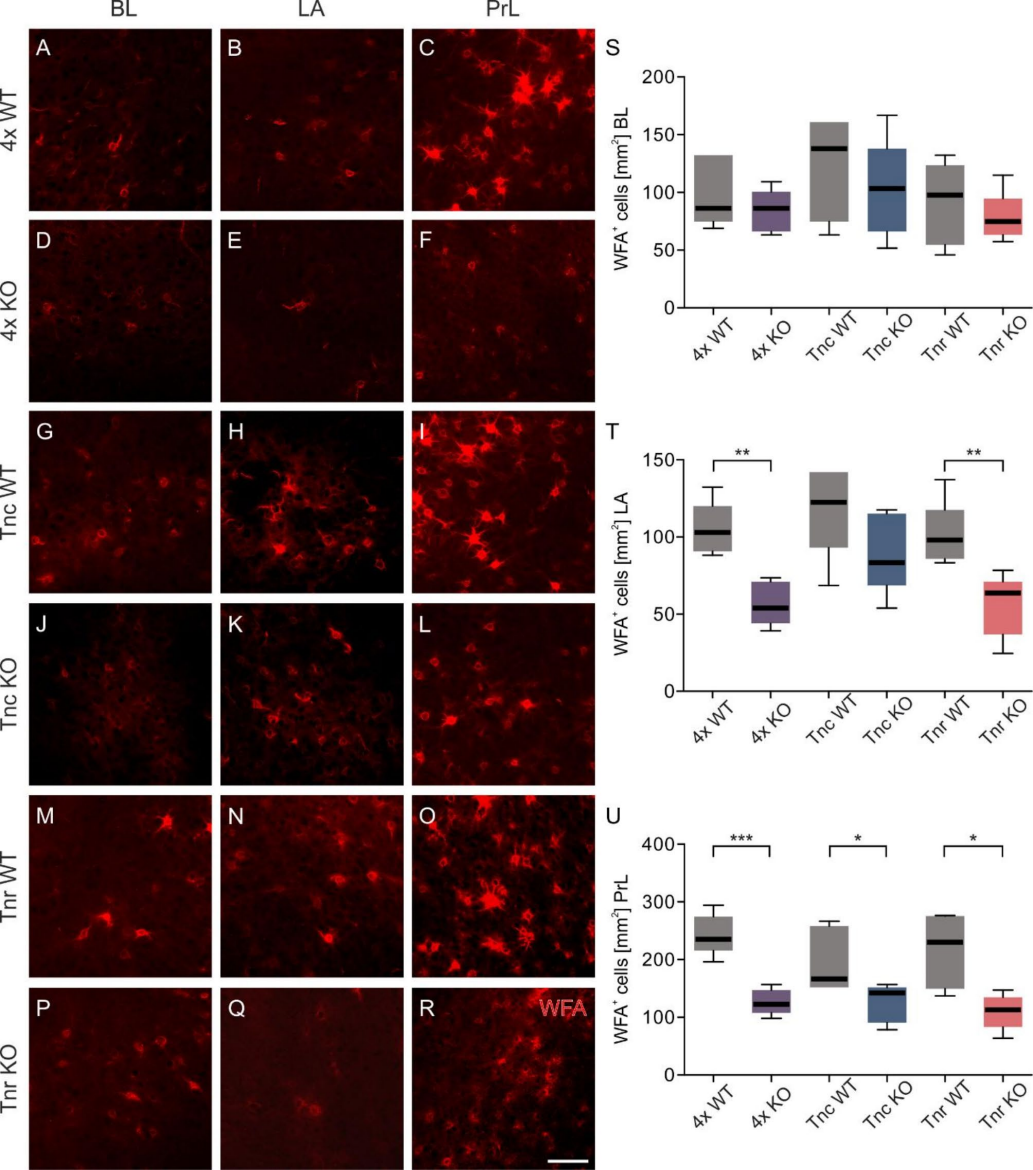
Reduction of perineuronal nets in tenascin C, tenascin R, brevican and neurocan deficient mice during cue retrieval. (**A**-**R**) PNNs in the PrL have a more complex structure with more WFA-positive proximal dendrites than PNNs in the BL and LA. The PNNs in the 4x KO mice lack the complexity in the PrL compared to the 4x WT mice. (**S**) The number of WFA-positive PNN enwrapped neurons in the BL is comparable in all examined genotypes. (**T**) The number of WFA-positive cells in the LA of 4x KO and Tnr KO mice was reduced in comparison to their control mice. (**U**) 4x KO, Tnc KO and Tnr KO mice showed less WFA-positive cells in the PrL in comparison to their control condition. PNN, perineuronal net; WFA, *Wisteria floribunda* agglutinin; ***p < 0.001; **p < 0.01; *p < 0.05; *N* = 5–6. scale bar = 50 μm.

To investigate whether the observed changes in fear behavior and neuronal activity in the corresponding brain areas are related to possible PNN alterations, the PNNs in these regions of the ECM KO animals were examined after cue retrieval. Based on the fluorescence microscopy images, clear differences were observed between the PNNs distribution of different genotypes and within the same genotype across specific fear associated brain regions (Fig. 4A-R). PNN-enveloped neurons were particularly abundant in the PrL of control lines, whereas the BL and LA contained less PNN populations. Interestingly, the statistical evaluation showed no differences in the number of PNNs in the BL of 4x KO, Tnc, or Tnr KO animals (Fig. 4S, Table S6) in comparison to their controls. On the other hand, direct consequences for the PNN populations in the LA from the 4x KO, as well as the Tnr single KO were detected. The number of PNNs in the LA of 4x KO (4x WT 105 ± 7.96 WFA^+^ cells/mm^2^ vs. 4x KO 56.8 ± 6.31 WFA^+^ cells/mm^2^; *p* ≤ 0.01; Fig. 4T) and Tnr single KO (Tnr WT 101 ± 9.50 WFA^+^ cells/mm^2^ vs. Tnr KO 55.9 ± 9.11 WFA^+^ cells/mm^2^; *p* ≤ 0.01; Fig. 4T) mice was reduced in comparison to their respective WT controls. In contrast, Tnc single KOs displayed comparable number of PNN-encased neurons in the LA (Table S6). The most pronounced effects on PNN population were observed in the PrL of ECM KOs. The number of WFA^+^ cells was strongly reduced in the PrL of the 4x KO mice (4x WT 243 ± 15.9 WFA^+^ cells/mm^2^ vs. 4x KO 126 ± 9.85 WFA^+^ cells/mm^2^; *p* ≤ 0.001; Fig. 4U) and to a lesser degree in the single Tnc KOs (Tnc WT 197 ± 25.1 WFA^+^ cells/mm^2^ vs. Tnc KO 125 ± 14.9 WFA^+^ cells/mm^2^; *p* ≤ 0.05; Fig. 4U) and Tnr KOs (Tnr WT 216 ± 28.7 WFA^+^ cells/mm^2^ vs. Tnr KO 110 ± 13.6 WFA^+^ cells/mm^2^; *p* ≤ 0.05; Fig. 4U) after cue retrieval. In summary, the 4x KOs and Tnc single KOs reduced PNN populations in LA after cue retrieval. Diminished PNN populations in the PrL were also evident in the 4x KO but also in the tenascin single KO mice.

Confocal laser-scanning microscopy suggests structural changes in the PNNs, in addition to changes in their number, from ECM KO animals. Since not only the number of PNNs but also the integrity of the individual net structures is crucial for the functionality of the enveloped neurons, super-resolution SIM images were analyzed to gain insights into the impact of the ECM KOs on PNN structure.

**Fig. 5:**
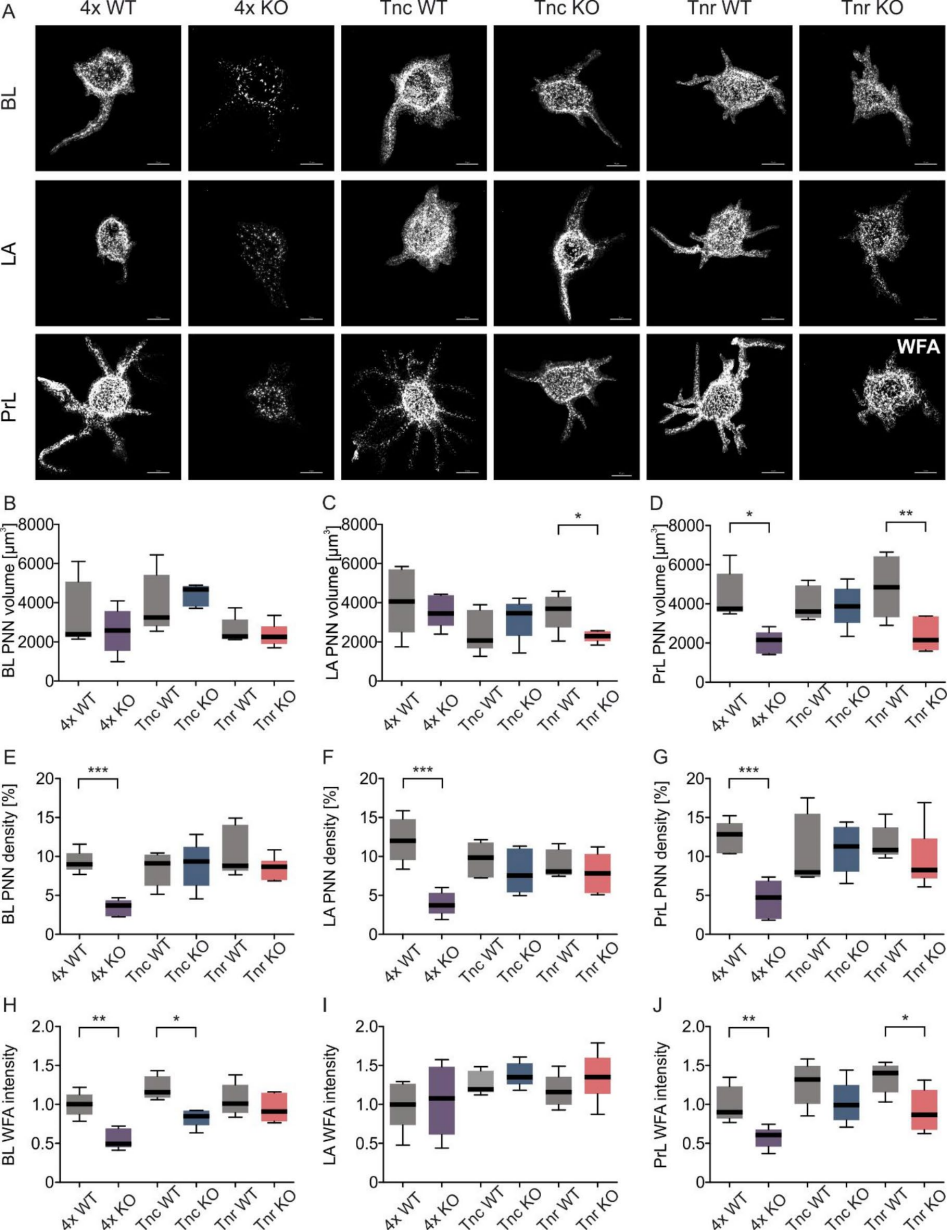
Disruption of perineuronal net structure in the BL, LA and PrL of tenascin-C, tenascin-R, brevican and neurocan deficient mice. (**A**) Representative SIM images of single PNNs in BL, LA and PrL of ECM KO and their control mice. (**B**) No differences of the PNN volume in BL of ECM-KO and their control mice. (**C**) Reduced PNN volume in the LA of Tnr KO mice in comparison to Tnr WT mice. (**D**) The PNN volume in the PrL of 4x KO and Tnr KO mice was decreased in comparison to their control animals. (**E-G**) Reduced PNN density in BL, LA and PrL of 4x KO mice in comparison to 4x WT mice. No alterations between the other genotypes. (**H**) Diminished WFA signal intensity in the BL of 4x KO and Tnc KO mice in comparison to their control animals. (**I**) Comparable WFA signal intensity between all examined genotypes in the LA. (**J**) Weak WFA signal intensity in the PrL of 4x KO and Tnr KO mice. ***p < 0.001; **p < 0.01; *p < 0.05; *N* = 5–6. scale bar = 50 μm.

The PNNs of control lines exhibited a very complex, characteristic honeycomb-like net structure with PNN-enveloped proximal dendrites in the PrL (Fig. 5A). In contrast the notably disrupted PrL morphology in the 4x KO, where isolated WFA^+^ signals were detected, formed neither a net-like structure nor surrounded the proximal dendrites. The PNN morphology also appeared disorganized in the PrL of Tnr KO animals. While a network structure was recognizable, fewer processes were PNN-enveloped compared to the Tnr WT PrL area. In the BL and LA, the PNN structure from the control lines does not appear as complex as in the PrL. This highlights the heterogeneity of PNNs in different CNS regions. Since the morphology of PNNs in these amygdala areas were not as complex, differences in PNN morphology in the ECM KOs were not as easily visible. However, the PNNs in the 4x KO clearly appeared disturbed. No differences in the BL and LA PNN volumes between genotypes were measured, except a reduction in the LA of Tnr KO mice (Fig. 5B and C). Decreased PNN volumes were also observed in the PrL of the 4x KO and Tnr KO animals (4x WT 4400 ± 551 PNN vol. [µm^3^] vs. 4x KO 2030 ± 262 [µm^3^]; *p* ≤ 0.01; Tnr WT 4870 ± 712 PNN vol. [µm^3^] vs. Tnr KO 2380 ± 334 [µm^3^]; *p* ≤ 0.01; Fig. 5D).

Since PNNs function as a physical barrier for synaptic connections, an impaired density can also affect the synaptic organization of the neuron they envelop^52,53^. This, in turn, can influence neuronal activity. Therefore, the density of PNNs was determined by calculating the proportion of WFA^+^ signal within the total volume of single PNNs. The density of the 4x KO PNNs were strongly disrupted in the BL (4x WT 9.3 ± 0.63% PNN density vs. 4x KO 3.42 ± 0.48 PNN density; *p* ≤ 0.001; Fig. 5E), the LA (4x WT 12.1 ± 1.28% PNN density vs. 4x KO 3.94 ± 0.68 PNN density; *p* ≤ 0.001; Fig. 5F) and the PrL (4x WT 12.4 ± 4.49% PNN density vs. 4x KO 4.49 ± 1.11 PNN density; *p* ≤ 0.001; Fig. 5G). In contrast, the tenascin single KOs showed no alterations in the PNN density in the examined areas (Fig. 5E-G, Table S6).

Furthermore, WFA intensity was significantly reduced in 4x KO animals in the BL compared to 4x WT (4x WT 1.0 ± 0.07 WFA intensity vs. 4x KO 0.56 ± 0.13 WFA intensity; *p* ≤ 0.01; Fig. 5H) and PrL (4x WT 1.0 ± 0.10 WFA intensity vs. 4x KO 0.57 ± 0.14 WFA intensity; *p* ≤ 0.01; Fig. 5J). Interestingly, Tnc KO mice showed also a reduced WFA intensity in the BL in comparison to Tnc WT mice (Tnc WT 1.21 ± 0.15 WFA intensity vs. Tnc KO 0.83 ± 0.11 WFA intensity; *p* ≤ 0.05; Fig. 5H). And Tnr KO mice showed reduced WFA intensity in the PrL in comparison to the Tnr WT (Tnr WT 1.34 ± 0.20 WFA intensity vs. Tnr KO 0.91 ± 0.27 WFA intensity; *p* ≤ 0.05; Fig. 5J). In contrast, WFA intensity in the LA was comparable between all genotypes (Fig. 5I, Table S6).

To analyze, if the disruption of PNNs also affects cells enwrapped by them, parvalbumin^+^ cells were immunohistochemically examined. Inhibitory fast-spiking parvalbumin^+^ interneurons represent the cell type most commonly enwrapped by PNNs. Therefore, the brain regions important for fear memory consolidation were also analyzed with respect to the number of parvalbumin^+^ and parvalbumin/WFA double-positive cells following cue retrieval (Figure S4). A significantly reduced number of parvalbumin^+^ cells (4x WT 45.80 ± 3.69 parvalbumin^+^ cells/mm^2^ vs. 4x KO 30.00 ± 4.90 parvalbumin^+^ cells/mm^2^; *p* ≤ 0.05; Figure S4K), as well as parvalbumin/WFA double-positive cells (4x WT 8.80 ± 1.74 parvalbumin^+^/WFA^+^ cells/mm^2^ vs. 4x KO 3.60 ± 1.29 parvalbumin^+^/WFA^+^ cells/mm^2^; *p* ≤ 0.05; Figure S4L), was observed in the PrL of 4x KO mice compared to 4x WT mice. In contrast, the number of parvalbumin^+^ and parvalbumin/WFA double-positive cells in BL and LA of 4x WT and 4x KO was comparable (Figure S4, Table S6).

Tnc KO and Tnr KO showed no differences in the number of parvalbumin^+^ and parvalbumin/WFA double-positive cells in comparison to their control animals in all observed brain areas (Figure S4, Table S6).

### Altered synaptic distribution along 4x KO PNNs is only partially due to tenascin R

**Fig. 6:**
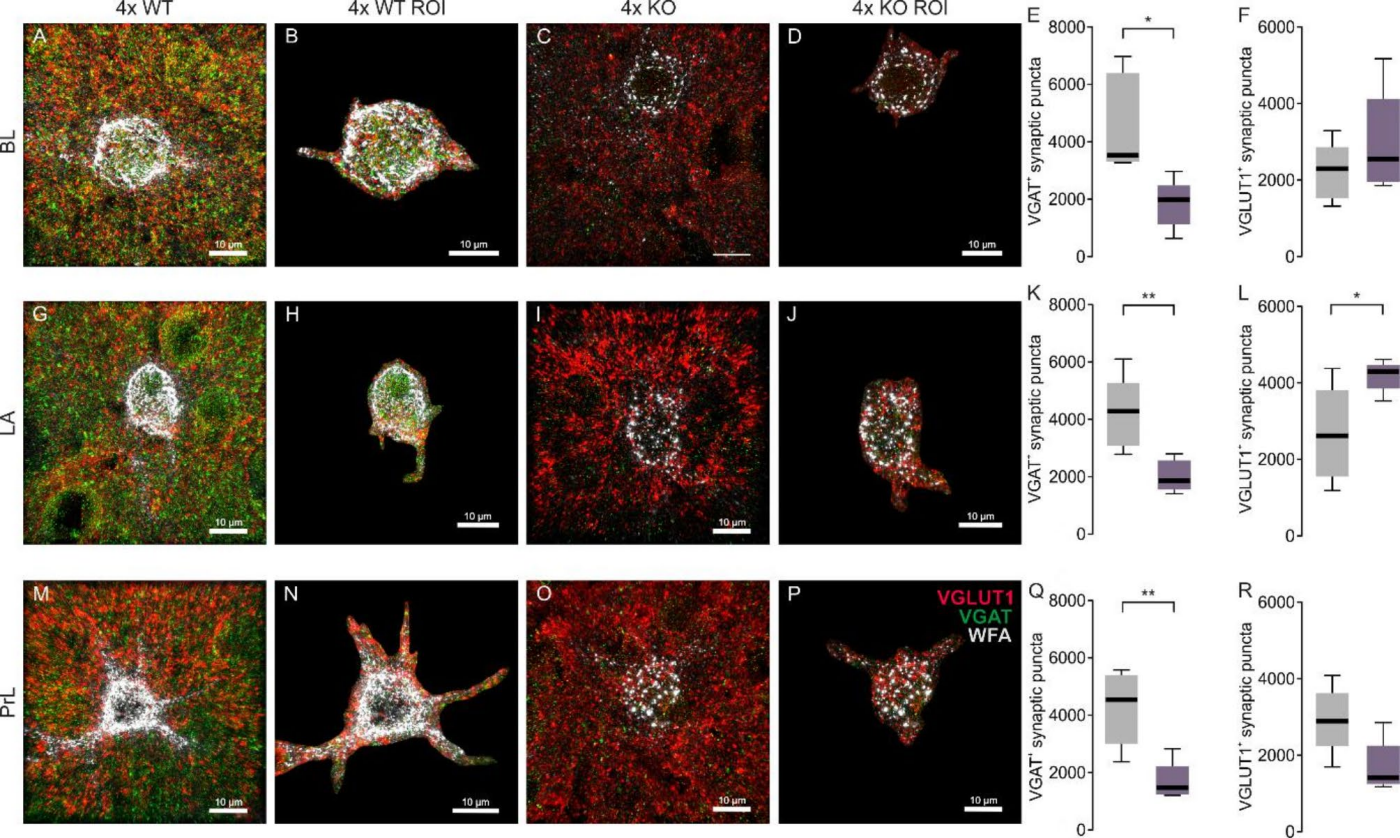
Reduced GABAergic signaling in brain areas important for the consolidation of fear in tenascin-C, tenascin-R, brevican and neurocan deficient mice during cue retrieval. (**A**-**D**, **G** & **J**, **M** & **P**) Distribution of VGAT^+^ (green) and VGLUT1^+^ (red) synaptic puncta along PNNs in BL, LA and PrL of 4x WT and 4x KO mice during cue retrieval. (**B**,** D**,** H**,** J**,** N**,** P**) ROI containing isolated PNNs with their synaptic distribution of VGAT^+^ and VGLUT1^+^ synaptic puncta. (**E**,** K**, **Q**) Reduction in the number of VGAT^+^ puncta in all examined brain areas of 4x KO mice in comparison to 4x WT mice. (**F**,** L**, **R**) Comparable number of VGLUT1^+^ synaptic puncta in BL, LA and PrL between 4x KO and 4x WT mice. ROI, region of interest; VGAT, vesicular GABA transporter; VGLUT1, vesicular glutamate transporter 1; **p < 0.01; *p < 0.05; *N* = 5–6. scale bar = 10 μm.

To investigate if disturbed PNN structure and number in the ECM KO mice has an effect on synaptic integrity, excitatory and inhibitory synapses on PNN encased neurons were analyzed^54,55^. PNNs influence the activity of the neurons they enclose by regulating the synaptic distribution along the soma and proximal dendrites. Therefore, super-resolution structured illumination microscopy (SIM) was used to analyze these presynaptic puncta (Figure S5). For this purpose, brain samples examined using WFA as a marker for PNNs, VGAT as a marker for inhibitory presynaptic structures, as well as VGLUT1 as a marker for excitatory presynaptic structures. In the BL, LA, and PrL regions of 4x WT and 4x KO mice, the synaptic puncta (“spots”) perforating the PNN (defined as ROI, region of interest) were analyzed using IMARIS (Fig. 6A-D, G-J, M-P). The synaptic organization in 4x KO mice exhibited less inhibitory VGAT^+^ puncta in the BL (4x WT 4580 ± 763 VGAT^+^ puncta/PNN vs. 4x KO 1840 ± 375 VGAT^+^ puncta/PNN; *p* ≤ 0.05; Fig. 6E), LA (4x WT 4200 ± 564 VGAT^+^ puncta/PNN vs. 4x KO 2020 ± 246 VGAT^+^ puncta/PNN; *p* ≤ 0.01; Fig. 6K) and PrL (4x WT 4340 ± 576 VGAT^+^ puncta/PNN vs. 4x KO 1750 ± 297 VGAT^+^ puncta/PNN; *p* ≤ 0.01; Fig. 6Q) compared to 4x WT mice after cue retrieval. In contrast, excitatory presynaptic structures were unaffected by the 4x KO. The number of VGLUT1^+^ puncta along 4x KO PNNs in the BL was comparable to VGLUT1^+^ puncta along 4x WT PNNs in the BL (Fig. 6F, Table S6). Interestingly, excitatory synaptic elements along PNNs in the LA of 4x KO mice were significantly increased in comparison to 4x WT mice after cue retrieval (4x WT 2620 ± 548 VGLUT1^+^ puncta/PNN vs. 4x KO 4140 ± 180 VGLUT1^+^ puncta/PNN; *p* ≤ 0.05; Fig. 6L). In the PrL, the number of VGLUT1^+^ puncta along PNNs was comparable between 4x KO and their control group (Fig. 6R, Table S6).

To investigate if tenascins are contributing to the alterations in synaptic distribution found in the 4x KO PNNs, we also examined the number of excitatory and inhibitory synaptic structures along PNNs in the fear associated brain regions of the amygdala and PrL from Tnc and Tnr KO mice after cue retrieval. The number of inhibitory VGAT^+^ puncta in the BL of Tnc KO mice was not significantly different from Tnc WT mice after cue retrieval (Figure S6E, Table S6), however substantially reduced in Tnr KO mice (Tnr WT 3680 ± 297 VGAT^+^ puncta/PNN vs. Tnr KO 2200 ± 259 VGAT^+^ puncta/PNN; *p* ≤ 0.01; Figure S7E). Similarly, the number of VGLUT1^+^ puncta along Tnr KO PNNs in the BL was decreased compared to Tnr WT PNNs (Tnr WT 1840 ± 298 VGLUT1^+^ puncta/PNN vs. Tnr KO 1100 ± 161 VGLUT1^+^ puncta/PNN; *p* ≤ 0.05; Figure S7F). No differences in the number of excitatory VGLUT1^+^ or inhibitory VGAT^+^ synaptic puncta along the LA PNNs from both Tnc and Tnr KO mice compared to their respective WT controls were found (Table S6). We also did not observe differences in the excitatory VGLUT1^+^ synapses along the PrL PNNs from both tenascin single KO lines (Tnc: Figure S6R; Tnr: Figure S7R) compared to their respective WT mice. However, the number of inhibitory VGAT^+^ synaptic puncta in the PrL of Tnr KO mice was significantly reduced compared to Tnr WT mice (Tnr WT 5680 ± 950 VGAT^+^ puncta/PNN vs. Tnr KO 3050 ± 291 VGAT^+^ puncta/PNN; *p* ≤ 0.05; Figure S7Q).

In summary, we found that impairment of PNNs impacts the synaptic integrity of their neurons. Removal of four PNN components led to an excitatory/inhibitory imbalance at the synapses along PNNs from the 4x KO mice, causing an overall reduced inhibitory and increased excitatory synaptic element. This synaptic imbalance may be partially due to the loss of Tnr, since inhibitory synapses were diminished in the Tnr KO BL and PrL and also in the excitatory synapses from the Tnr KO BL. However, the differences were not as pronounced as in the 4x KO. This could be due to the more severe disruption of PNNs in the 4x KO, suggesting an important role for the lecticans brevican and neurocan, which were not investigated in this study or an additive effect from a double tenascin KO. The observed less pronounced differences in the Tnr single KO may have been compensated by other PNN components, thereby potentially not sufficient to induce changes in fear consolidation. These synaptic changes may influence neuronal activity and downstream alterations in fear consolidation.

## Discussion

In the adult brain, synaptic plasticity is limited to maintain the stability of established neuronal networks^56,57^. This restriction is important for maintaining memory processes. A loss of this regulation can lead to impairments in memory processes and complex cognitive functions.

However, in conditions such as neurodegenerative diseases, PTSD, addiction or spinal cord injuries, restricted synaptic plasticity becomes disadvantageous^58–61^. This is because new neuronal connections cannot be formed to replace lost ones, preventing compensation, and leading to impaired neurological function^62^. Furthermore, restricted synaptic plasticity can hinder treatment of negative behavioral patterns like addiction or PTSD^63^ as inflexible neuronal connections can perpetuate these behaviors. One key factor that restricts synaptic plasticity is the maturation of PNNs^64^. PNNs inhibit synapse formation by acting as a physical barrier, binding synaptic inhibitors to specific sites on the CSPGs, or preventing the lateral diffusion of AMPA receptors^19,65,66^. Increasing evidence shows that the digestion of PNNs and the associated increase in synaptic plasticity are associated with short-term improvements in cognitive abilities or decreases in long-term memory, which may be beneficial for pathological behaviors such as PTSD or drug abuse^67–69^. However, the role of individual molecules and the possibility to target them individually for therapeutic interventions to prevent larger side effects is insufficiently studied.

The objective of this study was to evaluate the correlation between PNNs and memory processes in ECM mutant mice. We examined the structure, synaptic puncta and neuronal activity in brain areas contributing to the generation and maintenance of fear memories. ECM mutant mice were originally generated on the WT genetic background, which presents both challenges and potential opportunities if used for trauma-related research. These mice exhibit a high susceptibility to persisting fear memories as indicated by impaired fear extinction learning^70^. This persistent fear response may indicate low resilience making them a valuable model to study vulnerability to PTSD-like symptoms. Moreover, studies have demonstrated aberrant connectivity within the amygdala-medial prefrontal cortex circuit in the SV129 line^71^. This finding is consistent with human studies, which have similarly identified altered connectivity in this circuit among patients who suffer from PTSD^72,73^. The existence of such parallels serves to highlight the potential of SV129 mice as a starting point to apply changes to these PTSD-prone mice in order to further explore the PNN as a possible future treatment approach.

When 4x KO mice were subjected to a fear conditioning paradigm, they showed severe impairments in memory consolidation, as evidenced by their inability to retrieve the previously learned fear memory. Loss of fear consolidation was accompanied by reduced PNN density in all examined areas, suggesting a possible explanation for the observed memory impairment. PNN density is closely associated with the ability to maintain functional connections. While an increased density may lead to a loss of overall learning ability, the decrease in PNN density allows for better short term synapse formation but hinders long term memory maintenance^69,74^. Previous studies have shown, that increased PNN organization and changes in parvalbumin^+^ cells in the BL reduce plasticity and increase inhibition, which is accompanied by slower fear acquisition^75^.

The ECM KO animals studied, however, showed no abnormalities in the number of parvalbumin^+^ cells in the BL or LA. In contrast, 4x KO mice exhibited a reduced number of parvalbumin^+^ cells as well as PNN-enwrapped parvalbumin^+^ cells in the PrL.

In the PrL of 4x KO animals, the most severe impairments in PNN structure were observed. Their disruption could potentially affect these neurons. A reduced number of parvalbumin^+^ neurons has already been observed in the visual cortex of 4x KO mice^76^. Furthermore, post-training inhibition of parvalbumin^+^ cells in the prefrontal cortex or hippocampus has been shown to disrupt contextual fear memory consolidation^77^.

However, whether the reduced number of parvalbumin^+^ neurons in the 4x KO PrL also impacts fear memory in this model requires further investigation. This is particularly relevant given that no changes in cFOS expression were detected in the PrL of 4x KO animals, while changes were observed in the BL. It is worth noting that, in the amygdala, PNNs predominantly enwrap excitatory neurons rather than inhibitory parvalbumin^+^ neurons^78^.

Future studies should therefore examine the cell types enwrapped by PNNs in greater detail to determine whether PNN alterations differ between cell types in the ECM KOs.

Pantazopoulos et al. found a circadian PNN rhythm. They observed a decrease of PNNs during sleep and increased PNN levels during waking hours. By eliminating the PNN decrease by sleep deprivation after fear conditioning the researchers demonstrated that this process contributed to the consolidation of fear, underscoring the significance of dynamic, memory-related PNN changes^79^. In conclusion, the evidence indicates that PNN upregulation impairs the formation of new synapses whereas PNN downregulation enhances synapse formation but reduces the ability to consolidate these newly formed synapses.

The BL, an area crucially involved in retrieving and encoding fear memories^80^, displayed a reduction in cFOS intensity and a reduction in active, PNN-enwrapped neurons. This further indicates that the inability of 4x KO animals to retrieve fear memories seems to further support an impairment of memory consolidation.

Nevertheless, since no genotype differences in the number of cFOS-positive cells were observed in all examined brain regions, future studies should investigate expression of additional immediate early genes that are expressed during the fear conditioning test. Particularly, the activity-regulated cytoskeletal gene or protein would be of interest, as it is used as a molecular marker for the LA neuronal ensemble recruited during fear learning^81^.

Such changes in neural activity could be attributed to the observed reduction in VGAT^+^ synaptic puncta along the PNN-enwrapped cells, as the local inhibitory circuits in the amygdala are key players for the storage of fear memory^82^. This could further highlight the inability of PNN KO mice to stabilize GABAergic input. Furthermore, *in vitro studies* on 4x KO primary embryonic hippocampal neurons using whole-cell patch clamping already showed a reduced frequency of inhibitory post-synaptic- and excitatory post-synaptic currents compared to 4x WT primary embryonic hippocampal neurons^53^. Also, electrophysiological recordings of the spontaneous neuronal network activity of hippocampal neurons in vitro using multi electrode array measurements also showed an enhancement of the neuronal network activity level^54^. These findings were acompanied by alterations in the PNN structure and synaptic integrity.

These results are supported by a study that utilized the enzyme ChABC to degrade PNNs in the CA1 subregion of the hippocampus. This also impacted inhibitory synaptic transmission in CA1 neurons, accompanied by disrupted long term fear retrieval^83^. Furthermore, it has been demonstrated that the degradation of CSPGs in the amygdala by ChABC leads to the digestion of PNNs, rendering subsequently acquired fear memories particularly susceptible to erasure^38^. The infusion of PNN-digesting enzymes before cued fear conditioning in either the auditory cortex^84^ or mPFC^85^ resulted in the normal acquisition of fear memories, yet the retrieval of these memories was reduced. This further substantiates our conclusion that PNN disruption is prone to impair fear memory retrieval, potentially due to a lack of GABAergic input.

However, it should not be overlooked that the 4x KO animals showed less freezing, but also higher velocities compared to 4x WT mice. Therefore, the effects of ECM-KO on locomotion cannot be ruled out. Especially since it has already been shown that 4x KO animals exhibit impaired optomotor responses, which could at least partly be attributed to dysfunctions in visual processing^50^. Nevertheless, 4x KO animals should be subjected to intensive motor tests in the future to rule out motor impairments.

Erasing fear memories can be promising for individuals affected by PTSD. However, only 2% of CSPGs are bound to PNNs, and the majority are located in the interstitial matrix. Therefore, it is difficult to attribute these observed effects specifically to PNNs or even to individual PNN components. The 4x KO has already been shown to exhibit ECM alterations independent of the described PNN changes. The knockout of brevican, neurocan, tenascin-C, and tenascin-R leads to an upregulation of fibulin-1 and fibulin-2. Hyaluronan also showed altered expression^46^. Additionally, next-generation sequencing analyses of retinal tissues from 4x KO animals revealed changes in the expression of various ECM components, such as laminins, collagens, and diverse ECM receptors^50^.

This remodeling of the matrisome could, of course, also affect the behavior and memory of the animals. However, to our knowledge, none of the observed molecules, apart from the PNN components have been closely linked to memory performance thus far.

Single tenascin KOs, however, did not display any significant deficits in fear learning or memory, and histological changes were less conclusive. Slight alterations in PNNs and synaptic integrity were observed in the Tnr single KOs. However, these changes were not as pronounced as in the 4x KO indicating possible compensatory mechanisms. Especially in regard to cFOS activity and the overall density of the PNN network there were no changes which supports the hypothesis, that the density of the PNN is an important player in the maintenance of long-term memory. The profound effect in the 4x KO could also have been triggered by potential interactions between the four molecules. Interactions between tenascin-C and -R and the lecticans are well described^86^.

The 4x KO mutant refers to animals with a deletion of four genes simultaneously. Therefore, the observed changes in fear memory, PNN structure, neuronal activity, and synaptic integrity cannot be attributed to a single ECM protein. Since fear conditioning tests impose a high burden on the mice, only single KOs of the four genes with the most promising knowledge gain were chosen for this study. In this regard, Tnc and Tnr single KOs were selected because previous studies of visual behavior impairment in the 4x KO animals traced back to these single KOs^50^. It was observed that the simultaneous KO of the four genes has enhanced the effects. Since in this study, unlike the observed changes in the 4x KO, no differences were observed in the single KOs, future investigations should also test brevican and neurocan single KOs. Especially since both brevican and neurocan mRNA levels are dynamically regulated during auditory fear memory consolidation^84^. Additionally, neurocan secreted by astrocytes controls the formation of inhibitory synapses^87^.

## Electronic supplementary material

Below is the link to the electronic supplementary material.

Supplementary Material 1

## Data Availability

The datasets generated during and/or analyzed during the current study are available from the corresponding author upon reasonable request.

## Abbreviations

BL: Basolateral amygdala
BSA: Bovine serum albumin
ChABC: Chondroitinase ABC
CNS: Central nervous system
CSPG: Chondroitin sulfate proteoglycan
ECM: Extracellular matrix
GABA: Gamma-aminobutyric acid
HAPLN: Hyaluronan and proteoglycan link protein
KO: Knockout
min: minutes
PrL: Limbic cortex
mPFC: Medial prefrontal cortex
Otx2: Orthodenticle homeobox 2
PBS: Phosphate-buffered saline
PFA: Paraformaldehyde
PNN: Perineuronal net
RT-qPCR: Quantitative real time polymerase chain-reaction
PSD95: Postsynaptic density protein 95
ROI: Region of interest
RSC: Retrosplenial cortex
SD: Standard deviation
SEM: Standard error mean
SIM: Structured illumination microscopy
Tnc: Tenascin-C
Tnr: Tenascin-R
V1: Primary visual cortex
VGAT: Vesicular GABA transporter
VGLUT1: Vesicular glutamate transporter 1
WFA: Wisteria floribunda agglutinin
WT: Wild-type
