# Supplementary material for: Combined loss of brevican, neurocan, tenascin-C and tenascin-R leads to impaired fear retrieval due to perineuronal net loss
Source: Sci Rep. 2025 Feb 14;15:5528. doi: 10.1038/s41598-025-89580-2 (PMC11828866; doi:10.1038/s41598-025-89580-2)
Supplement: Supplementary file 1 — Supplementary Material 1 [file 41598_2025_89580_MOESM1_ESM.docx]

# Supplementary

### Table S1: Antibodies for immunohistochemical staining

| Primary  antibody/Lectin | Species,  Clonality/type | Dilution | Source/Research  Resource Identifier  (RRID) | Secondary  antibody | Species | Dilution/source |
| --- | --- | --- | --- | --- | --- | --- |
| cFOS | Rabbit,  IgG,  polyclonal | 1:500 | Synaptic Systems GmbH; AB_2891278 | Anti-  Rabbit  Cy2 | Goat | 1:400 Dianova |
| Parvalbumin | Chicken, IgY, polyclonal | 1:200 | Synaptic Systems GmbH;  AB_2619887 | Anti-  Chicken  Cy2 | Goat | 1:400 Dianova |
| VGLUT1 | Guinea pig, IgG, polyclonal | 1:300 | Synaptic Systems GmbH; AB_887878 | Anti-guinea pig Cy5 | Goat | 1:400 Dianova |
| VGAT | Guinea pig, polyclonal, IgG | 1:300 | Synaptic Systems GmbH; AB_887873 | Anti-guinea pig Cy5 | Goat | 1:400 Dianova |
| Wisteria floribunda  agglutinin | Lectin | 1:200 | Vector Laboratories; AB_2336874 | Streptavidin Cy3 Cy2 |  | 1:400 Dianova |

### Table S2. Results of the two-way repeated measures mixed-effect analysis for freezing behavior between mice groups during fear acquisition.

| ***n***  ***WT/ KO*** | **Factor** | **Sphericity** | **Num Df** | **Den Df** | ***F*** | ***P*** |
| --- | --- | --- | --- | --- | --- | --- |
| **Fear acquisition** | | | | | | |
| *fear acquisition training Tnr WT/ Tnr KO* | | | | | | |
| 6/6 | Trial  Genotype  Trial × Genotype | Greenhouse-Geisser corrected epsilon 0.2937 | 2.643  1  9 | 26.43  10  90 | 40.45  0.013  1.491 | **<.001**  .912  .163 |
| *fear acquisition training Tnc WT/ Tnc KO* | | | | | | |
| 8/8 | Trial  Genotype  Trial × Genotype | Greenhouse-Geisser corrected epsilon 0.4105 | 3.695  1  9 | 51.73  14  126 | 32.13  1.146  0.3852 | **<.001**  .302  .940 |
| *fear acquisition training* 4x KO 4x WT */ 4x KO* | | | | | | |
| 14/14 | Trial  Genotype  Trial × Genotype | Greenhouse-Geisser corrected epsilon 0.4864 | 4.378  1  9 | 113.8  26  234 | 93.60  0.7297  1.936 | **<.001**  .401  **.048** |
|  | | | | | | |

### Table S3. Results of the two-way repeated measures mixed-effect Analysis for freezing behavior during fear retrieval.

| ***Genotype*** | **Factor** | **Num Df** | **Den Df** | ***F*** | ***P*** |
| --- | --- | --- | --- | --- | --- |
| *fear retrieval* Tnr WT/ Tnr KO | | | | | |
| 6/6 | Trial (Baseline x Retrieval)  Genotype  Trial × Genotype | 1  1  1 | 5  5  5 | 55.46  2.427  1.517 | **<.001**  0.180  0.273 |
| **Post-*hoc* test**   \| **Genotype** \| **Trial** \| ***p-Value Tukey adjusted*** \| \| --- \| --- \| --- \| \| Tnr WT \| Baseline x Retrieval \| **0.002** \| \| Tnr KO \| Baseline x Retrieval \| **0.049** \| \| Tnr WT x Tnr KO \| Baseline \| 0.740 \| \| Tnr WT x Tnr KO \| Retrieval \| 0.672 \|  \| *fear retrieval* Tnc WT / Tnc KO \| \| \| \| \| \| \| \| --- \| --- \| --- \| --- \| --- \| --- \| --- \| \| 8/8 \| Trial (Baseline x Retrieval)  Genotype  Trial × Genotype \| 1  1  1 \| 7  7  7 \| 29.93  0.014  0.026 \| **<.001**  0.907  0.877 \| \| **Post-hoc test**   \| **Genotype** \| **Trial** \| ***p-Value Tukey adjusted*** \| \| --- \| --- \| --- \| \| Tnc WT \| Baseline x Retrieval \| **0.032** \| \| Tnc KO \| Baseline x Retrieval \| **0.019** \| \| Tnc WT x Tnc KO \| Baseline \| 0.755 \| \| Tnc WT x Tnc KO \| Retrieval \| >.999 \|  \| *fear retrieval 4x* WT/ 4x KO \| \| \| \| \| \| \| --- \| --- \| --- \| --- \| --- \| --- \| \| 14/14 \| Trial (Baseline x Retrieval)  Genotype  Trial × Genotype \| 1  1  1 \| 13  13  13 \| 268.3  79.30  644.3 \| **<.001**  **<.001**  **<.001** \| \| **Post-hoc test**   \| **Genotype** \| **Trial** \| ***p-Value Tukey adjusted*** \| \| --- \| --- \| --- \| \| 4x WT \| Baseline x Retrieval \| **< .001** \| \| 4x KO \| Baseline x Retrieval \| 0.343 \| \| WT x 4x KO \| Baseline \| 0.160 \| \| WT x 4x KO \| Retrieval \| **< .001** \| \| \| \| \| \| \|   * Significant results at *p* < 0.05.  *** Significant results at *p* < 0.001. \| \| \| \| \| \| \| | | | | | |

### Table S4. Results of the two-way repeated measures mixed-effect analysis for velocity during fear retrieval.

| ***Genotype*** | **Factor** | **Num Df** | **Den Df** | ***F*** | ***P*** |
| --- | --- | --- | --- | --- | --- |
| *fear retrieval* Tnr WT / Tnr KO | | | | | |
| 6/6 | Trial (Baseline x Retrieval)  Genotype  Trial × Genotype | 1  1  1 | 5  5  5 | 97.67  0.738  0.003 | **<.001**  0.430  0.961 |
| **Post-*hoc* test**   \| **Genotype** \| **Trial** \| ***p-Value Tukey adjusted*** \| \| --- \| --- \| --- \| \| Tnr WT \| Baseline x Retrieval \| **0.003** \| \| Tnr KO \| Baseline x Retrieval \| **0.004** \| \| Tnr WT x Tnr KO \| Baseline \| 0.933 \| \| Tnr WT x Tnr KO \| Retrieval \| 0.775 \|  \| *fear retrieval* Tnc WT/ Tnc KO \| \| \| \| \| \| \| \| --- \| --- \| --- \| --- \| --- \| --- \| --- \| \| 8/8 \| Trial (Baseline x Retrieval)  Genotype  Trial × Genotype \| 1  1  1 \| 7  7  7 \| 120.4  0.265  0.711 \| **<.001**  0.623  0.427 \| \| **Post-hoc test**   \| **Genotype** \| **Trial** \| ***p-Value Tukey adjusted*** \| \| --- \| --- \| --- \| \| Tnc WT \| Baseline x Retrieval \| **<.001** \| \| Tnc KO \| Baseline x Retrieval \| **0.002** \| \| Tnc WT x Tnc KO \| Baseline \| 0.910 \| \| Tnc WT x Tnc KO \| Retrieval \| >.999 \|  \| *fear retrieval* WT/ 4x KO KO \| \| \| \| \| \| \| \| --- \| --- \| --- \| --- \| --- \| --- \| --- \| \| 14/14 \| Trial (Baseline x Retrieval)  Genotype  Trial × Genotype \| 1  1  1 \| 13  13  13 \| 186  32.87  5.414 \| **<.001**  **<.001**  **0.037** \| \| **Post-hoc test**   \| **Genotype** \| **Trial** \| ***p-Value Tukey adjusted*** \| \| --- \| --- \| --- \| \| 4x WT \| Baseline x Retrieval \| **<.001** \| \| 4x KO \| Baseline x Retrieval \| **<.001** \| \| WT x 4x KO \| Baseline \| **0.003** \| \| WT x 4x KO \| Retrieval \| **<.001** \| \| \| \| \| \| \| \| \| \| \| \| \| \| \| | | | | | |

### Table S5. Results of the two-way repeated measures mixed-effect analysis for freezing behavior between 4x KO and 4x WT during the extended fear conditioning protocol.

| ***n***  ***WT/ KO*** | | | **Factor** | | **Sphericity** | | **Num Df** | | **Den Df** | | ***F*** | | ***P*** | |  |
| --- | --- | --- | --- | --- | --- | --- | --- | --- | --- | --- | --- | --- | --- | --- | --- |
| **Fear acquisition** | | | | | | | | | | | | | | |  |
| *fear acquisition training* 4x KO WT */ 4x KO* | | | | | | | | | | | | | | |  |
| 6/6 | | | Trial  Genotype  Trial × Genotype | | Greenhouse-Geisser corrected epsilon 0.4168 | | 3.751  1  9 | | 37.51  10  90 | | 35.99  2.733  1.032 | | **<.001**  .129  .421 | |  |
| *fear extinction I* 4x WT */ 4x KO* | | | | | | | | | | | | | | |  |
| 6/6 | | | Trial  Genotype  Trial × Genotype | | Greenhouse-Geisser corrected epsilon 0.3267 | | 4.574  1  14 | | 45.74  10  140 | | 1.296  22.38  0.5195 | | .284  **<.001**  .919 | |  |
| *fear extinction II* 4x WT */ 4x KO* | | | | | | | | | | | | | | |  |
| 6/6 | | | Trial  Genotype  Trial × Genotype | | Greenhouse-Geisser corrected epsilon 0.3876 | | 5.427  1  14 | | 54.27  10  140 | | 6.131  24.77  2.359 | | **<.001**  **<.001**  **.006** | |  |
| fear extinction III 4x WT / 4x KO | | | | | | | | | | | | | | |  |
| 6/6 | | Trial  Genotype  Trial × Genotype | | Greenhouse-Geisser corrected epsilon 0.2737 | | 3.831  1  14 | | 38.31  10  140 | | 3.814  28.54  2.434 | | **.011**  **<.001**  **.004** | |  |  |
| *fear extinction IV* 4x WT */ 4x KO* | | | | | | | | | | | | | | | |
| 6/6 | | | | Trial  Genotype  Trial × Genotype | | Greenhouse-Geisser corrected epsilon 0.2827 | | 3.958  1  14 | | 39.58  10  140 | | 2.245  10.73  1.694 | | **.**082  **.008**  **.063** | |
| *fear extinction II* 4x WT */ 4x KO* | | | | | | | | | | | | | | | |
| 6/6 | | | | Trial  Genotype  Trial × Genotype | | Greenhouse-Geisser corrected epsilon 0.3579 | | 5.011  1  14 | | 50.11  10  140 | | 6.686  31.71  2.563 | | **<.001**  **<.001**  **.003** | |

### Table S6. Overview of the collected values of the immunohistochemical staining against cFOS and the synaptic markers.

| ***Marker*** | **Brain area** | **Genotype** | **value** | ***Statistical test*** |
| --- | --- | --- | --- | --- |
| cFOS^+^ cells | BL | 4x Wt  4x KO  Tnc WT  Tnc KO  Tnr WT  Tnr KO | 289 ± 13.8 cells/mm^2^  349 ± 32.2 cells/mm^2^  224 ± 52.1 cells/mm^2^  130 ± 16.4 cells/mm^2^  240 ± 44.2 cells/mm^2^  190 ± 34.2 cells/mm^2^ | **Unpaired t-test** |
| cFOS^+^ cells | LA | 4x Wt  4x KO  Tnc WT  Tnc KO  Tnr WT  Tnr KO | 105 ± 7.96 cells/mm^2^  56.8 ± 6.31 cells/mm^2^  119 ± 13.5 cells/mm^2^  90 ± 11.6 cells/mm^2^  101 ± 9.5 cells/mm^2^  55.9 ± 9.1 cells/mm^2^ | **Unpaired t-test** |
| cFOS^+^ cells | PrL | 4x Wt  4x KO  Tnc WT  Tnc KO  Tnr WT  Tnr KO | 781 ± 150 cells/mm^2^  690 ± 160 cells/mm^2^  529 ± 64.1 cells/mm^2^  332 ± 91.4 cells/mm^2^  626 ± 106 cells/mm^2^  564 ± 42 cells/mm^2^ | **Unpaired t-test** |
| parvalbumin^+^  cells | BL | 4x Wt  4x KO  Tnc WT  Tnc KO  Tnr WT  Tnr KO | 11.00 ± 2.63 cells/mm^2^  18.2 ±2.11 cells/mm^2^  18.17 ± 3.11 cells/mm^2^  20.00 ± 2.12 cells/mm^2^  15.33 ± 2.99 cells/mm^2^  17.10 ± 3.01 cells/mm^2^ | **Unpaired t-test** |
| parvalbumin^+^ cells | LA | 4x Wt  4x KO  Tnc WT  Tnc KO  Tnr WT  Tnr KO | 16.6 ± 3.09 cells/mm^2^  14.2 ± 4.03 cells/mm^2^  18.83 ± 3.94 cells/mm^2^  26.2 ± 5.63 cells/mm^2^  23.50 ± 3.47 cells/mm^2^  19.67 ± 3.97 cells/mm^2^ | **Unpaired t-test** |
| parvalbumin^+^ cells | PrL | 4x Wt  4x KO  Tnc WT  Tnc KO  Tnr WT  Tnr KO | 45.8 ± 3.69 cells/mm^2^  30.0 ± 3.22 cells/mm^2^  35.0 ± 5.27 cells/mm^2^  39.4 ± 2.49 cells/mm^2^  33.00 ± 2.97 cells/mm^2^  25.70 ± 2.44 cells/mm^2^ | **Unpaired t-test** |
| parvalbumin^+^/WFA^+^ cells | BL | 4x Wt  4x KO  Tnc WT  Tnc KO  Tnr WT  Tnr KO | 2.80 ± 0.80 cells/mm^2^  0.80 ± 0.58 cells/mm^2^  2.2 ± 0.68 cells/mm^2^  3.0 ± 0.70 cells/mm^2^  3.02 ± 0.77 cells/mm^2^  3.33 ± 0.88 cells/mm^2^ | **Mann-Whitney test** |
| parvalbumin^+^/WFA^+^ cells | LA | 4x Wt  4x KO  Tnc WT  Tnc KO  Tnr WT  Tnr KO | 2.60 ± 0.67 cells/mm^2^  1.80 ± 0.66 cells/mm^2^  2.83 ± 0.91 cells/mm^2^  3.60± 0.75 cells/mm^2^  2.20 ± 0.85 cells/mm^2^  1.10 ± 0.51 cells/mm^2^ | **Mann-Whitney test** |
| parvalbumin^+^/WFA^+^ cells | PrL | 4x Wt  4x KO  Tnc WT  Tnc KO  Tnr WT  Tnr KO | 8.80 ± 1.74 cells/mm^2^  3.60 ± 1.29 cells/mm^2^  7.50 ± 1.61 cells/mm^2^  7.40 ± 1.33 cells/mm^2^  7.00 ± 0.73 cells/mm^2^  3.33 ± 0.58 cells/mm^2^ | **Unpaired t-test** |
| mean cFOS^+^  intensity | BL | 4x Wt  4x KO  Tnc WT  Tnc KO  Tnr WT  Tnr KO | 0.67 ± 0.03  0.39 ± 0.04  0.62 ± 0.04  0.50 ± 0.07  0.50 ± 0.08  0.55 ±0.08 | **Unpaired t-test** |
| mean cFOS^+^  intensity | LA | 4x Wt  4x KO  Tnc WT  Tnc KO  Tnr WT  Tnr KO | 0.54 ± 0.06  0.54 ± 0.09  0.51 ± 0.08  0.42 ± 0.08  0.49 ± 0.07  0.45 ±0.07 | **Unpaired t-test** |
| mean cFOS^+^  intensity | PrL | 4x Wt  4x KO  Tnc WT  Tnc KO  Tnr WT  Tnr KO | 0.56 ± 0.05  0.42 ± 0.03  0.41 ± 0.02  0.38 ± 0.03  0.52 ± 0.06  0.46 ±0.05 | **Unpaired t-test** |
| WFA^+^ cells | BL | 4x Wt  4x KO  Tnc WT  Tnc KO  Tnr WT  Tnr KO | 100 ± 13.4 cells/mm^2^  83.9 ± 8.3 cells/mm^2^  122 ± 20 cells/mm^2^  102 ± 19.0 cells/mm^2^  90.8 ± 16 cells/mm^2^  78.2 ± 9.7 cells/mm^2^ | **Unpaired t-test** |
| WFA^+^ cells | LA | 4x Wt  4x KO  Tnc WT  Tnc KO  Tnr WT  Tnr KO | 105 ± 7.7 cells/mm^2^  56.8 ± 6.3 cells/mm^2^  119 ± 13.5 cells/mm^2^  90.2 ± 11.6 cells/mm^2^  101 ± 9.5 cells/mm^2^  55.9 ± 9.1 cells/mm^2^ | **Unpaired t-test** |
| WFA^+^ cells | PrL | 4x Wt  4x KO  Tnc WT  Tnc KO  Tnr WT  Tnr KO | 243 ± 15.9 cells/mm^2^  126 ± 9.8 cells/mm^2^  197 ± 25.1 cells/mm^2^  125 ± 14.9 cells/mm^2^  216 ± 28.7 cells/mm^2^  110 ± 13.6 cells/mm^2^ | **Unpaired t-test** |
| mean WFA^+^  intensity | BL | 4x Wt  4x KO  Tnc WT  Tnc KO  Tnr WT  Tnr KO | 1.00 ± 0.07  0.56 ± 0.06  1.21 ± 0.07  0.84 ± 0.05  1.06 ± 0.09  0.95 ±0.07 | **Unpaired t-test** |
| mean WFA^+^  intensity | LA | 4x Wt  4x KO  Tnc WT  Tnc KO  Tnr WT  Tnr KO | 1.00 ± 0.44  1.05 ± 0.21  1.28 ± 0.07  0.38 ± 0.07  1.17 ± 0.09  1.35 ±0.13 | **Unpaired t-test** |
| mean WFA^+^  intensity | PrL | 4x Wt  4x KO  Tnc WT  Tnc KO  Tnr WT  Tnr KO | 1.00 ± 0.10  0.57 ± 0.06  1.27 ± 0.12  1.02 ± 0.07  1.34 ± 0.09  0.92 ±0.11 | **Unpaired t-test** |
| WFA & cFOS  double positive cells | BL | 4x Wt  4x KO  Tnc WT  Tnc KO  Tnr WT  Tnr KO | 27.4 ± 4.3 cells/mm^2^  16.7 ± 1.2 cells/mm^2^  14.7 ± 1.8 cells/mm^2^  17.6 ± 3.3 cells/mm^2^  16.7 ± 5.5 cells/mm^2^  19.6 ± 4.4 cells/mm^2^ | **Unpaired t-test** |
| WFA & cFOS  double positive cells | LA | 4x Wt  4x KO  Tnc WT  Tnc KO  Tnr WT  Tnr KO | 11.8 ± 2.5 cells/mm^2^  14.7 ± 2.7 cells/mm^2^  15.5 ± 4.3 cells/mm^2^  19.6 ± 4.7 cells/mm^2^  15.7 ± 2.9 cells/mm^2^  16.3 ± 4.9 cells/mm^2^ | **Unpaired t-test** |
| WFA & cFOS  double positive cells | PrL | 4x Wt  4x KO  Tnc WT  Tnc KO  Tnr WT  Tnr KO | 59.8 ± 14.6 cells/mm^2^  25.5 ± 3.3 cells/mm^2^  28.6 ± 5.6 cells/mm^2^  22.5 ± 5.9 cells/mm^2^  34.3 ± 4.4 cells/mm^2^  35.1 ± 5.7 cells/mm^2^ | **Unpaired t-test** |
| PNN volume [µm^3^] | BL | 4x Wt  4x KO  Tnc WT  Tnc KO  Tnr WT  Tnr KO | 3410 ± 756  2560 ± 514  3940 ± 697  4400 ± 247  2570 ± 301  2350 ± 236 | **Unpaired t-test** |
| PNN volume [µm^3^] | LA | 4x Wt  4x KO  Tnc WT  Tnc KO  Tnr WT  Tnr KO | 4090 ± 759  3570 ± 376  2530 ± 480  3190 ± 473  3560 ± 425  2270 ± 118 | **Unpaired t-test** |
| PNN volume [µm^3^] | PrL | 4x Wt  4x KO  Tnc WT  Tnc KO  Tnr WT  Tnr KO | 4400 ± 551  2030 ± 262  4010 ± 392  3890 ± 464  4870 ± 712  2380 ± 334 | **Unpaired t-test** |
| PNN density [%] | BL | 4x Wt  4x KO  Tnc WT  Tnc KO  Tnr WT  Tnr KO | 9.3 ± 0.63  3.4 ± 0.48  8.4 ± 0.98  8.9 ± 1.34  10.7 ± 1.43  8.51 ±0.60 | **Unpaired t-test** |
| PNN density [%] | LA | 4x Wt  4x KO  Tnc WT  Tnc KO  Tnr WT  Tnr KO | 12.1 ± 1.28  3.9 ± 0.68  9.6 ± 1.01  8.1 ± 1.28  9.0 ± 0.81  7.9 ±1.06 | **Unpaired t-test** |
| PNN density [%] | PrL | 4x Wt  4x KO  Tnc WT  Tnc KO  Tnr WT  Tnr KO | 12.4 ± 0.99  4.5 ± 1.11  10.7 ± 2.05  11.0 ± 1.39  11.7 ± 0.99  9.6 ±1.58 | **Unpaired t-test** |
| VGAT^+^ synaptic puncta | BL | 4x Wt  4x KO  Tnc WT  Tnc KO  Tnr WT  Tnr KO | 4580 ± 736  1840 ± 839  3960 ± 676  4080 ± 247  3680 ± 297  2200 ± 259 | **Unpaired t-test** |
| VGAT^+^ synaptic puncta | LA | 4x Wt  4x KO  Tnc WT  Tnc KO  Tnr WT  Tnr KO | 4200 ± 564  2020 ± 246  4590 ± 1160  2570 ± 470  4110 ± 795  2340 ± 292 | **Unpaired t-test** |
| VGAT^+^ synaptic puncta | PrL | 4x Wt  4x KO  Tnc WT  Tnc KO  Tnr WT  Tnr KO | 4340 ± 576  1750 ± 297  4990 ± 406  5260 ± 1100  5680 ± 950  3050 ± 291 | **Unpaired t-test** |
| VGLUT1^+^ synaptic puncta | BL | 4x Wt  4x KO  Tnc WT  Tnc KO  Tnr WT  Tnr KO | 2210 ± 336  2940 ± 597  2620 ± 238  2330 ± 374  1840 ± 298  1100 ± 161 | **Unpaired t-test** |
| VGLUT1^+^ synaptic puncta | LA | 4x Wt  4x KO  Tnc WT  Tnc KO  Tnr WT  Tnr KO | 2620 ± 548  4140 ± 180  2000 ± 280  2240 ± 504  2300 ± 643  1300 ± 141 | **Unpaired t-test** |
| VGLUT1^+^ synaptic puncta | PrL | 4x Wt  4x KO  Tnc WT  Tnc KO  Tnr WT  Tnr KO | 2930 ± 383  1690 ± 303  2250 ± 278  3220 ± 815  1960 ± 140  1480 ± 288 | **Unpaired t-test** |


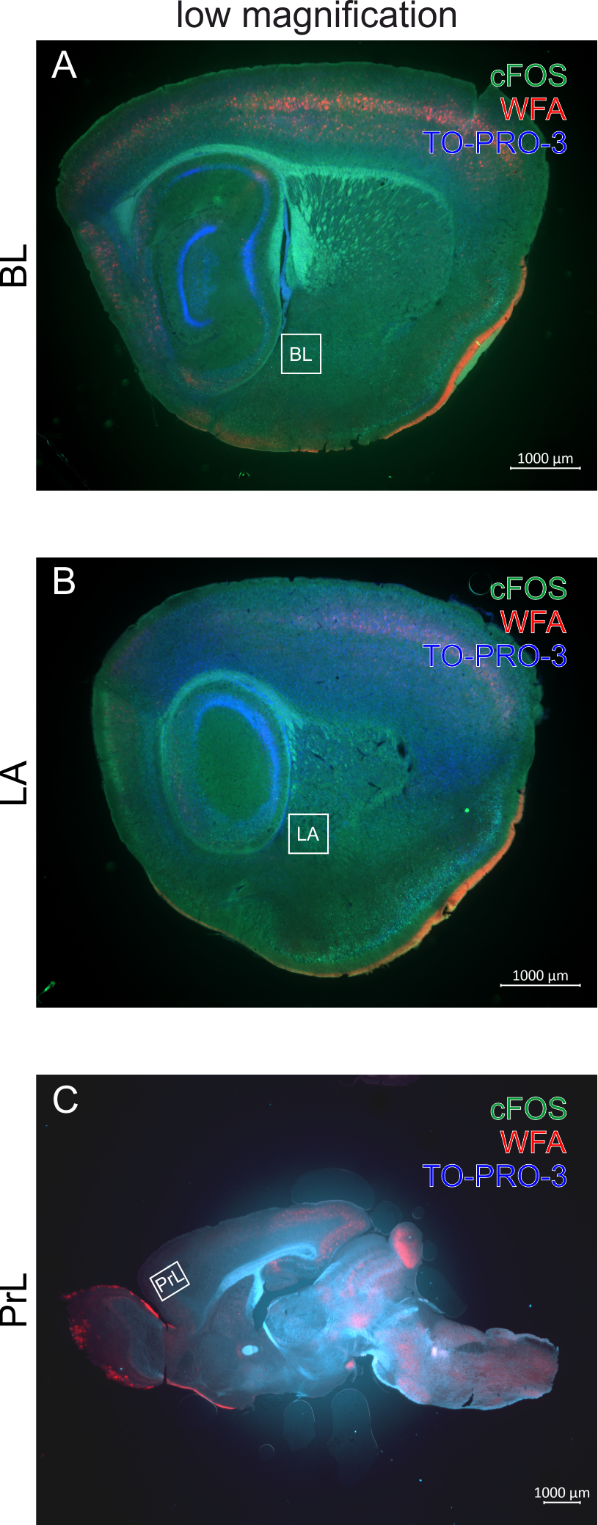


Figure S1. **Overview images of the examined brain regions at low magnification.** **(A-C)** To ensure comparable imaging of the immunohistochemically analyzed regions BL, LA, and PrL, overview images of the sagittal brain sections were acquired beforehand. cFOS immunoreactivity was displayed in green, WFA immunoreactivity in red, and TO-PRO-3 in blue. scale bar = 1000 µm


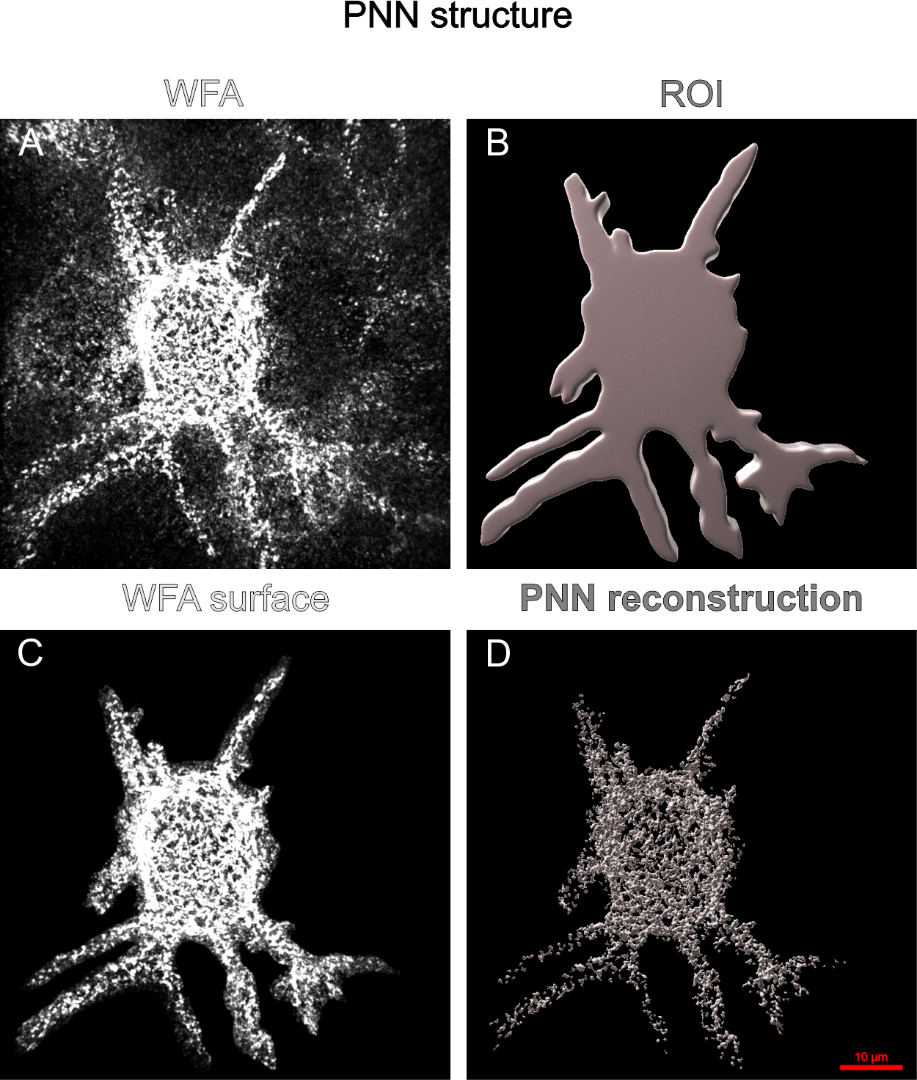


Figure S2. **Evaluation of the PNN structure in BL, LA und PrL of ECM KO mice after cue retrieval using SIM.** **(A)** Representative super-resolution SIM image of a 4x WT PNN located in the PrL. The lattice like structure and the PNN-encased proximal dendrites are clearly recognizable. **(B)** Region of interest containing the PNN generated *via* IMARIS software. **(C)** A WFA surface generated within the ROI. Signal outside the ROI was dismissed. **(D)** PNN reconstruction was achieved with IMARIS surface technology. Properties such as volume, intensity and density of the PNN can be displayed and determined through the reconstruction. PNN, perineuronal net; ROI, region of interest; WFA, *wisteria floribunda* agglutinin; scale bar = 10 µm


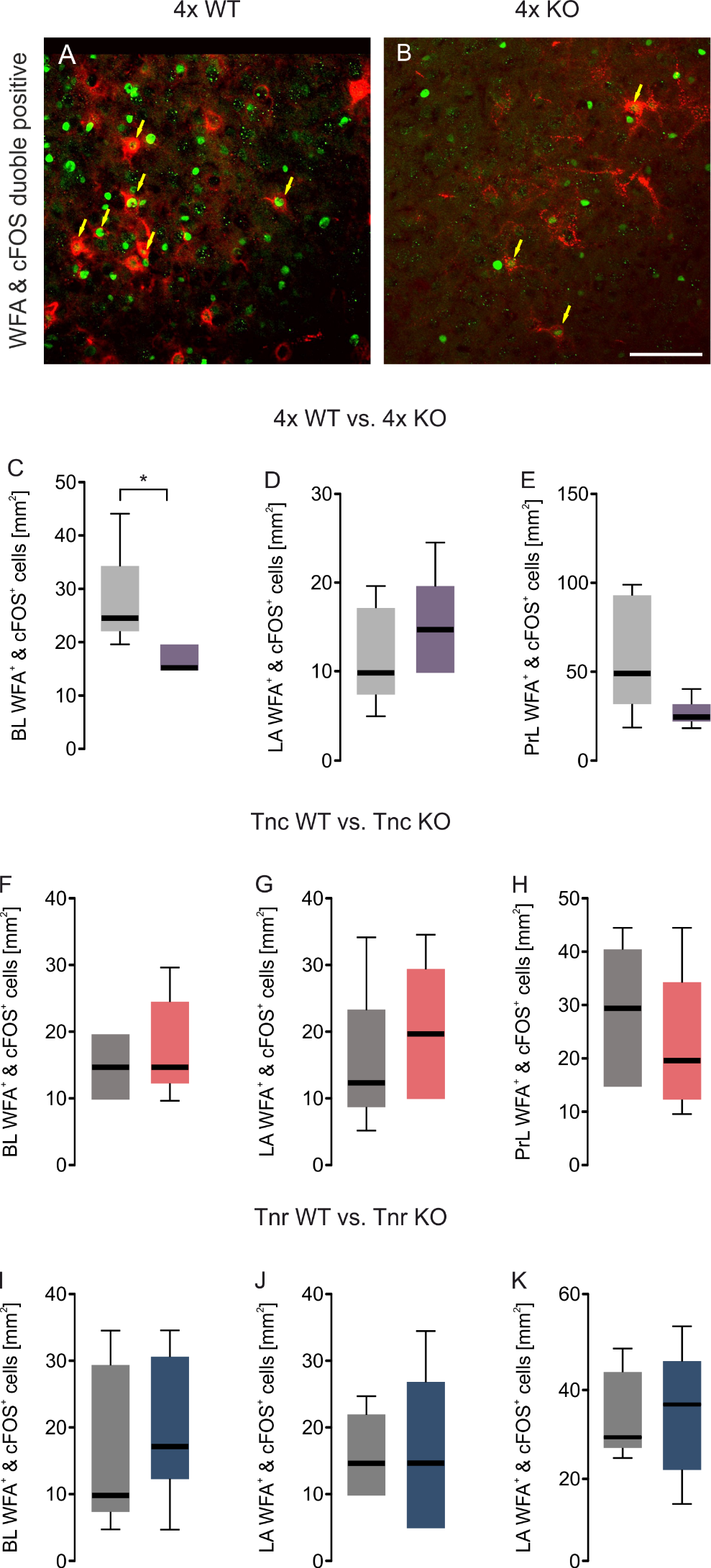


Figure S3. **Reduced number of neuronal active PNN-enveloped cells in the BL of 4x KO mice after cue retrieval. (A & B)** Exemplary images of WT and 4x KO WFA and cFOS double positive cells (yellow arrows) in the BL. **(C)** The number of WFA and cFOS double positive cells was significantly reduced in the BL of 4x KO mice in comparison to 4x WT mice after cue retrieval. **(D & E)** The number of WFA and cFOS double positive cells in the LA and PrL was comparable between 4x KO and 4x WT mice after cue retrieval. **(F- H)** No significant differences in the number of WFA and cFOS double positive cells was observed in BL, LA and PrL between Tnc KO and Tnc WT mice after cue retrieval. **(I- K)** The number of WFA and cFOS double positive cells was comparable in BL, LA and PrL between Tnr WT and KO animals after cue retrieval. BL, basolateral amygdala; LA, lateral amygdala; PrL, prelimbic cortex; N=5-6. scale bar = 50 µm


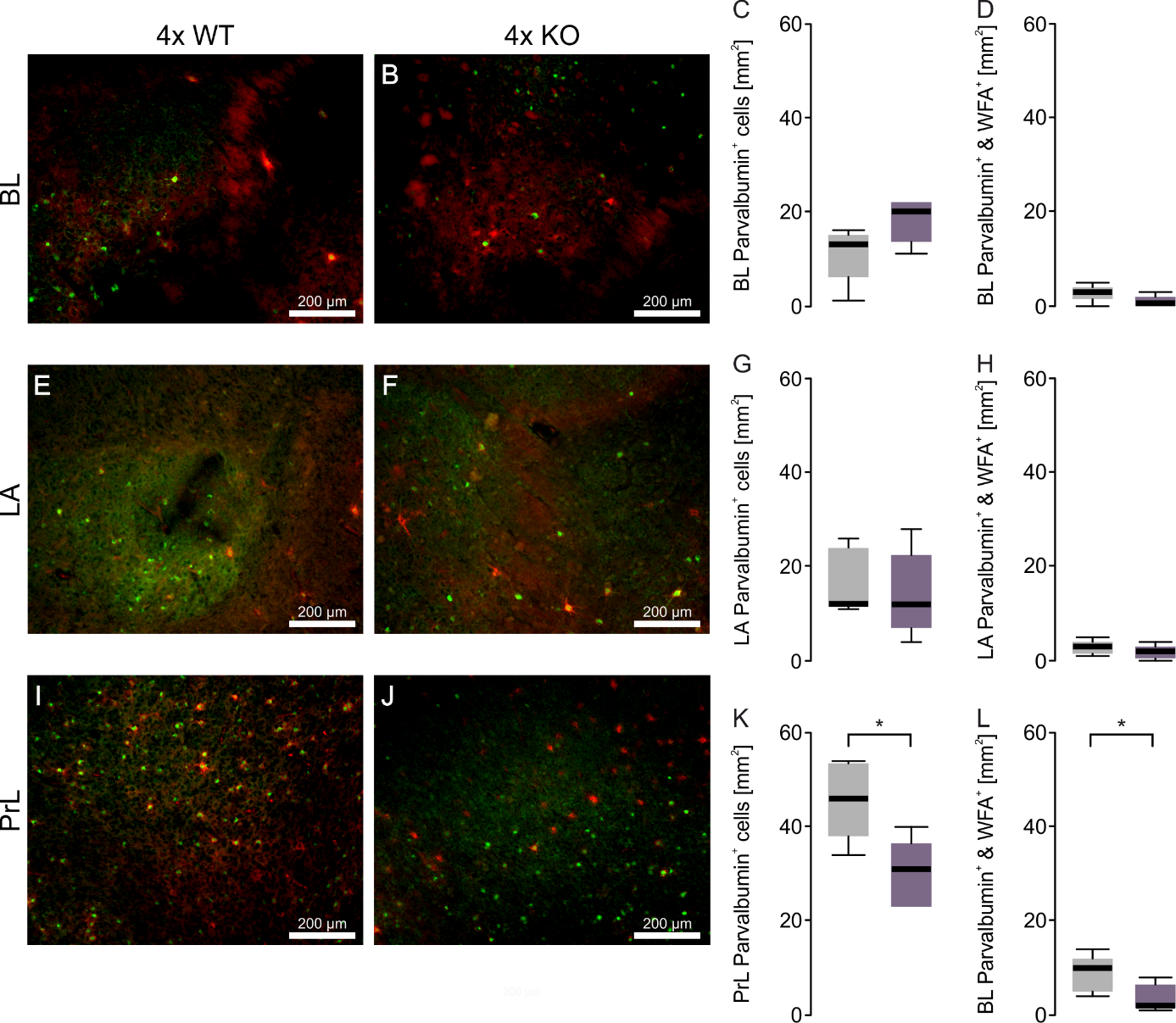


Figure S4. **Reduced number of parvalbumin^+^ cells in the PrL of 4x KO mice after cue retrieval. (A & B, E & F, I & J)** Exemplary images of WT and 4x KO WFA^+^ (red) and parvalbumin^+^ positive cells in the BL, LA and PrL of 4x WT and 4x KO mice after cue retrieval. **(C & D, G & H)** The number of parvalbumin^+^ and parvalbumin/WFA double positive cells was comparable in the BL and LA of 4x KO mice in comparison to 4x WT mice after cue retrieval. **(D & E)** The number of WFA and cFOS double positive cells in the LA and PrL was comparable between 4x KO and 4x WT mice after cue retrieval. **(K & L)** The number of parvalbumin^+^ cells was significantly reduced in the PrL of 4x KO mice in comparison to the 4x WT. Furthermore, the number of WFA+ PNN encased parvalbumin+ cells was significantly reduced in the 4x KO PrL; N=5. scale bar = 200 µm


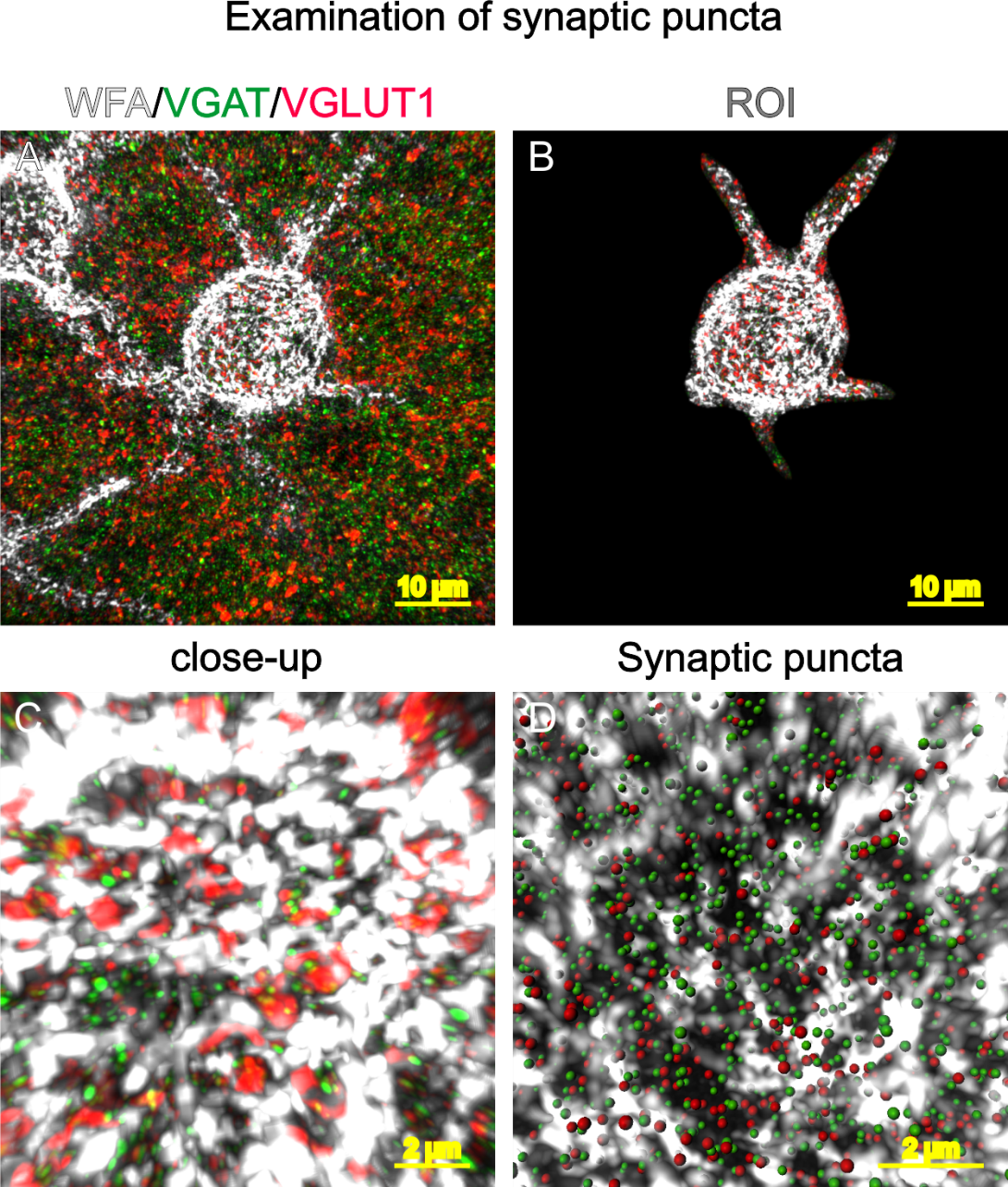


Figure S5. **Examination of the synaptic distribution in BL, LA und PrL of ECM KO mice along PNNs *via* super-resolution SIM. (A)** Representative image of a PNN and presynaptic puncta. Antibodies against VGAT (green) and VGLUT1 (red) were used as marker for presynaptic puncta and WFA (white) as marker for PNNs. **(B)** A ROI around the PNN was generated and signal outside the ROI was suppressed using IMARIS software to analyze presynaptic distribution along the PNN. **(C)** A close-up of the ROI shows the VGAT^+^ (green) and VGLUT1^+^ (red) synaptic puncta perforating the holes of the PNN. **(D)** Immunopositive signal of VGAT and VGLUT1 were identified as synaptic puncta and visualized as spots, matching an estimated puncta size of 0.2 μm and fitting the default intensity threshold. Immunoreactive signals that did not meet these criteria were dismissed as background noise. The number of spots was automatically calculated by the IMARIS software. VGAT = vesicular GABA transporter, VGLUT1 = vesicular glutamate transporter 1, WFA = Wisteria floribunda agglutinin, scale bar A & B = 10 µm, scale bar C & D = 2 µm


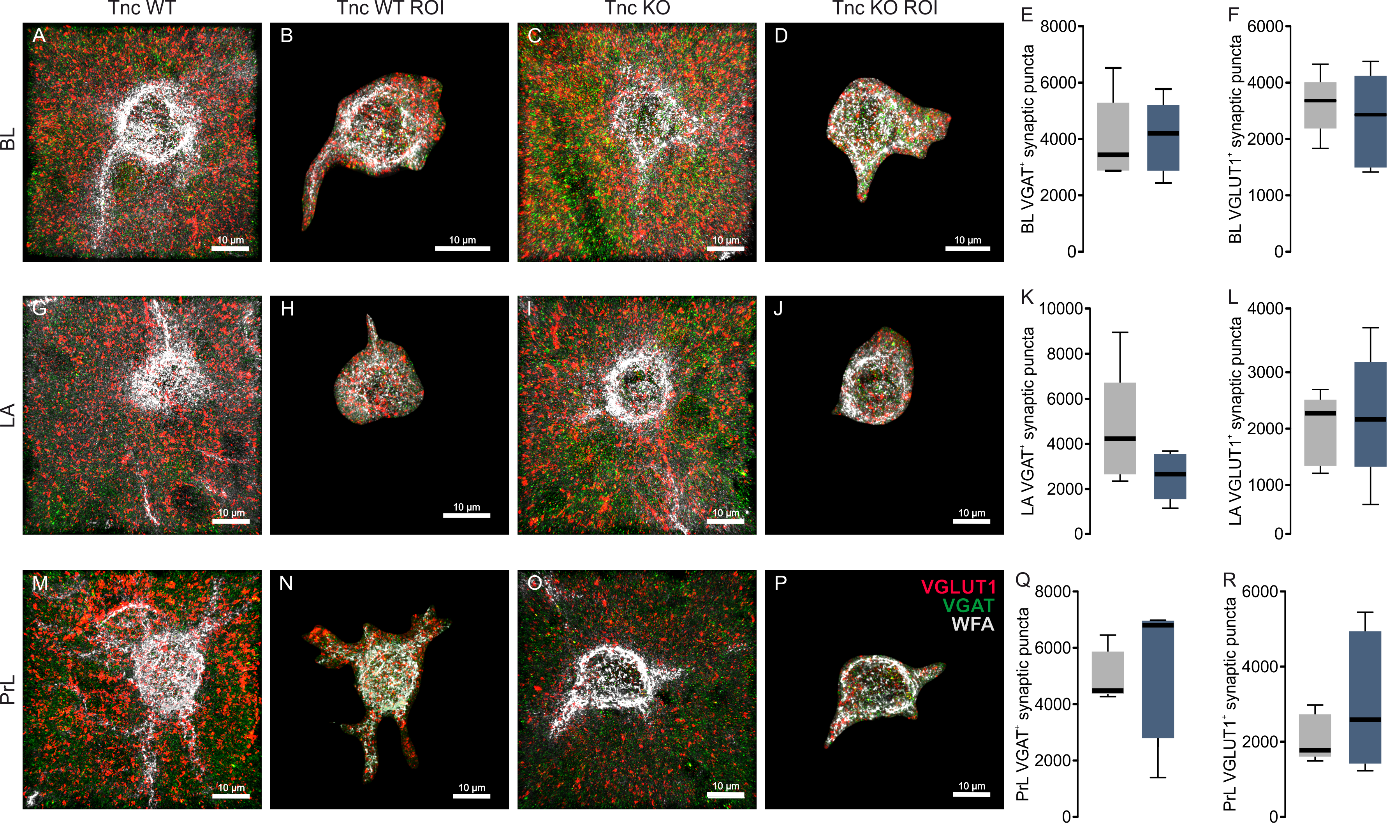


**Figure S6. Comparable GABAergic and glutamatergic signaling in brain areas important for the consolidation of fear in *tenascin-C* deficient mice during cue retrieval. (A-D, G-J, M-P)** Distribution of VGAT^+^ (green) and VGLUT1^+^ (red) synaptic puncta along PNNs in BL, LA and PrL of Tnc WT and Tnc KO mice during cue retrieval. **(B, D, H, J, N, P)** ROI containing isolated PNNs with their synaptic distribution of VGAT^+^ and VGLUT1^+^ synaptic puncta. **(E, K, Q)** Comparable number of VGAT^+^ puncta in all examined brain areas of Tnc KO in comparison to Tnc WT mice. **(F, L, R)** Comparable number of VGLUT1^+^ synaptic puncta in BL, LA and PrL between Tnc KO and Tnc WT mice.
N=5-6. scale bar = 10 µm


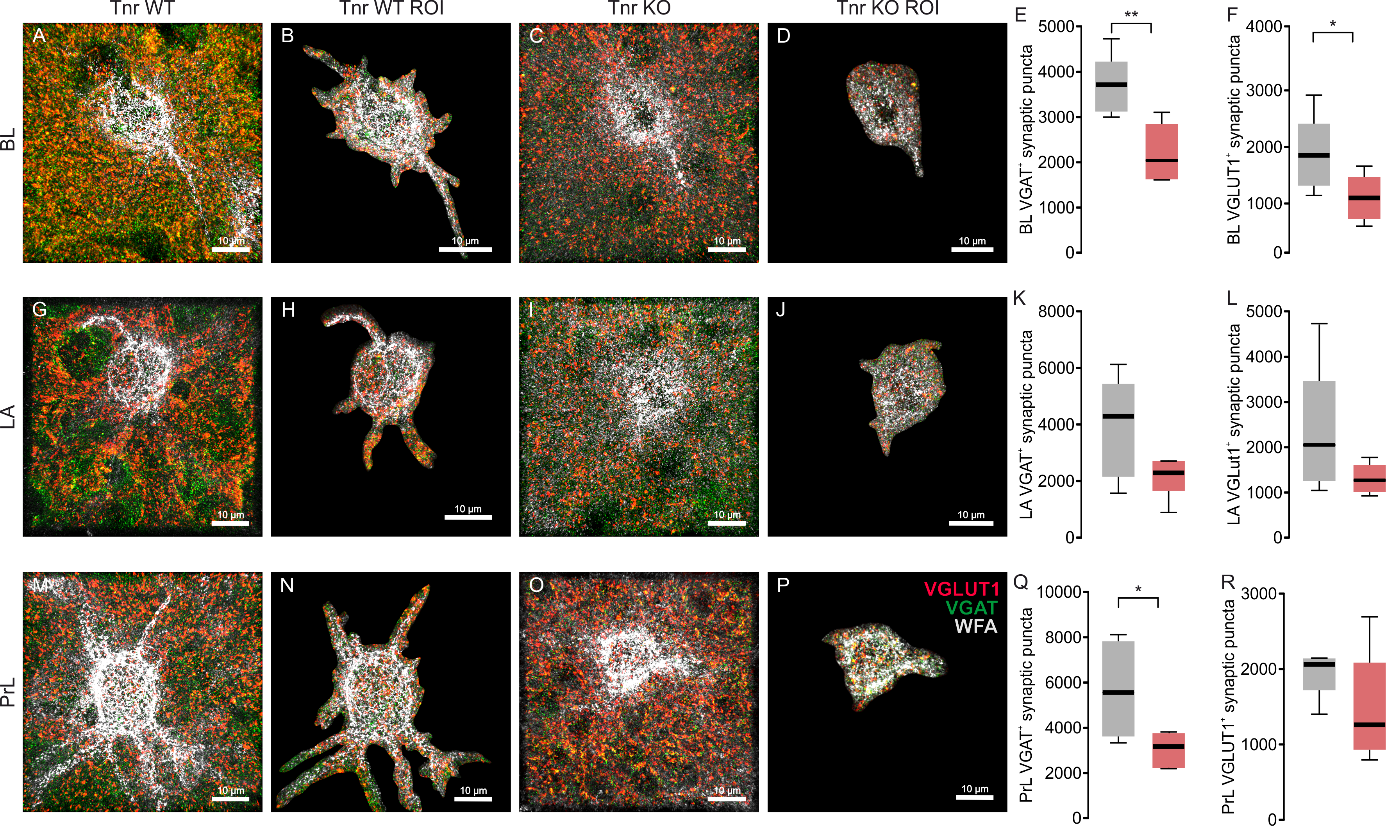


**Figure S7. Reduced synaptic signaling in BL and PrL areas important for the consolidation of fear in *tenascin-R* deficient mice during cue retrieval. (A-D, G-J, M-P)** Distribution of VGAT^+^ (green) and VGLUT1^+^ (red) synaptic puncta along PNNs in BL, LA and PrL of Tnr WT and Tnr KO mice during cue retrieval. **(B, D, H, J, N, P)** ROI containing isolated PNNs with their synaptic distribution of VGAT^+^ and VGLUT1^+^ synaptic puncta. **(E&Q)** Significantly reduced number of VGAT^+^ synaptic puncta in BL and PrL of Tnr KO mice in comparison to Tnr WT mice. **(K)** Comparable number of VGAT^+^ puncta in LA of Tnr KO in comparison to Tnr WT mice. **(F)** Reduced number of VGLUT1^+^ puncta in the BL of Tnr KO mice in comparison to Tnr WT mice. **(L&R)** Comparable number of VGLUT1^+^ synaptic puncta in LA and PrL between Tnr KO and Tnr WT mice.
***p* < 0.01; **p* < 0.05; N=5-6. scale bar = 10 µm


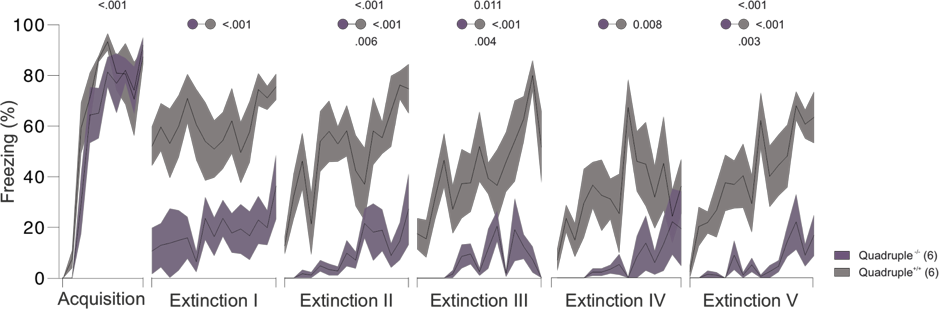


**Figure S8. Mice of the WT strain display impaired fear extinction learning while 4x KO KO animals demonstrate persistent lack of fear towards the conditioning stimulus during extinction.** The extended fear conditioning paradigm consisted of an acquisition phase where a tone co-terminated with a shock six times to archive a fearful association to the tone**.** 4x KO KO and WT mice exhibited increasing time spent freezing during the conditioning stimulus. Forty-eight h after conditioning, mice were subjected to fear extinction learning, where the conditioning stimulus was played 14 times to extinguish the previously learned fear memory. The extinction session was repeated on five consecutive days. Comparisons of the time spent freezing during the acquisition revealed that both lines showed significant increases in freezing. All extinction sessions demonstrated significant differences between the groups, while extinction days 2, 3, and 5 revealed an interaction effect between the groups and the course of the extinction session (detailed statistics in Table S5). The data are presented as the mean (line) ± SEM (shaded area). The number of animals tested is shown in parentheses behind the genotype description. The p-value displayed at the top of the graph depicts the course of the behavior during the session. The second p-value refers to the group effect, while the p-value displayed at the bottom of the graph represents the interaction effect of the two values displayed above it. N=5-6.
